# The role of heme in sepsis induced Kupffer cell PANoptosis and senescence

**DOI:** 10.1038/s41419-025-07637-6

**Published:** 2025-04-13

**Authors:** Tingting Li, Joseph Adams, Peilin Zhu, Tao Zhang, Fei Tu, Amy Gravitte, Xiaojin Zhang, Li Liu, Jared Casteel, Valentin Yakubenko, David L. Williams, Chuanfu Li, Xiaohui Wang

**Affiliations:** 1 Department of Biomedical Sciences, Quillen College of Medicine, East Tennessee State University, Johnson City, TN 37614 USA; 2 UMPC Hillman Cancer Center, University of Pittsburgh, Pittsburgh, PA 15232 USA; 3 Department of Geriatrics, Jiangsu Provincial Key Laboratory of Geriatrics, The First Affiliated Hospital of Nanjing Medical University, Nanjing, 210029 China; 4 Center of Excellence in Inflammation, Infectious Disease, and Immunity, Quillen College of Medicine, East Tennessee State University, Johnson City, TN 37614 USA; 5 Department of Surgery, Quillen College of Medicine, East Tennessee State University, Johnson City, TN 37614 USA

**Keywords:** Cell biology, Immunology

## Abstract

Elevated heme levels, a consequence of hemolysis, are strongly associated with increased susceptibility to bacterial infections and adverse sepsis outcomes, particularly in older populations. However, the underlying mechanisms remain poorly understood. Using a cecal ligation and puncture (CLP) model of sepsis, we demonstrate that elevated heme levels correlate with Kupffer cell loss, increased bacterial burden, and heightened mortality. Mechanistically, we identify mitochondrial damage as a key driver of heme- and bacterial-induced Kupffer cell PANoptosis, a form of cell death integrating pyroptosis, apoptosis, and necroptosis, as well as cellular senescence. Specifically, heme activates phospholipase C gamma (PLC-γ), facilitating the translocation of cleaved gasdermin D (c-GSDMD) to mitochondria, resulting in GSDMD pore formation, mitochondrial dysfunction, and the release of mitochondrial DNA (mtDNA) during bacterial infection. This mitochondrial damage amplifies PANoptosis and triggers the cGAS-STING signaling pathway, further driving immune senescence. Notably, PLC-γ inhibition significantly reduces mitochondrial damage, cell death, and senescence caused by heme and bacterial infection. Furthermore, we show that hemopexin, a heme scavenger, effectively mitigates sepsis-induced Kupffer cell death and senescence, enhances bacterial clearance, and improves survival outcomes in both young and aged mice. These findings establish mitochondrial damage as a central mediator of heme induced Kupffer cell loss and highlight PLC-γ inhibition and hemopexin administration as promising therapeutic strategies for combating sepsis associated immune dysfunction.

## Introduction

Sepsis, defined as a dysregulated immune response to infection, remains a leading cause of death in ICUs, particularly among elderly individuals [1–3]. In the United States, sepsis accounts for approximately 1.7 million cases annually, causing nearly 350,000 deaths [4, 5]. Sepsis associated immune dysfunction is a key contributor to poor survival and secondary infections. However, the underlying mechanisms remain incompletely understood.

Kupffer Cells, the resident macrophages of the liver and the largest population of tissue resident macrophages, play a pivotal role in eliminating invading pathogens, clearing damaged cells, and removing debris from the bloodstream [6–9]. This process is critical for preventing disseminated infections and limiting organ damage, particularly during systemic infections such as sepsis. Impaired Kupffer cell function has been linked to increased susceptibility to bacterial infections and poor sepsis outcomes [7, 10–12]. In this study, we observed severe Kupffer cell death and senescence during sepsis, which correlated with increased bacterial load and heightened mortality, particularly in aged mice. However, the mechanisms driving Kupffer cell death and senescence during sepsis remain largely unexplored.

Hemolysis, a common complication of sepsis, leads to the release of free heme, a potent pro-oxidant and pro-inflammatory damage associated molecular pattern (DAMP) [13–17]. Elevated free heme levels have been associated with increased sepsis severity, as heme disrupts endothelial barrier integrity [18], impairs bacterial clearance [19, 20], and contributes to multi-organ damage [21]. Recent in vitro studies suggest that heme promotes pathogen-associated molecular patterns (PAMPs) induced PANoptosis, an integrated cell death pathway encompassing pyroptosis (P), apoptosis (A), and necroptosis (N), by enhancing the formation of NLRP12 and NLRC5 mediated PANoptosome complex [14, 22]. However, whether elevated heme contributes to sepsis associated Kupffer cell death remains undefined. In this study, we used a mouse sepsis model to demonstrate that elevated heme levels are strongly correlated with sepsis associated Kupffer cell death. Distinct from previous reports, we identify mitochondrial damage as a central driver of heme induced cell death. Specifically, heme exposure significantly exacerbates bacterial infection induced mitochondrial damage, further amplifying GSDMD-mediated pyroptosis, RIPK/MLKL-mediated necroptosis, and caspase-mediated apoptosis. Additionally, the release of mitochondrial DNA (mtDNA) activates the cGAS-STING signaling pathway, driving macrophage senescence [23, 24]. Cellular senescence, characterized by cell cycle arrest and the acquisition of a proinflammatory senescence-associated secretory phenotype (SASP) [25, 26], represents another potential mechanism of Kupffer cell dysfunction, impairing their ability to self-renew and effectively respond to infection.

Our mechanistic studies demonstrate that heme activates phospholipase C gamma (PLC-γ), facilitating the translocation of cleaved gasdermin D (cGSDMD) to mitochondria, which leads to GSDMD pore formation and mitochondrial damage. Notably, PLC-γ inhibition alleviates heme-and-bacterial induced cell death and senescence. Furthermore, our in vivo studies show that increased expression of hemopexin, a heme scavenger [27], significantly alleviates sepsis-induced Kupffer cell death and senescence, improving survival outcomes in both young and aged mice.

## Results

### Sepsis induced Kupffer cell loss correlates with increased bacterial load and higher mortality

**Fig. 1:**
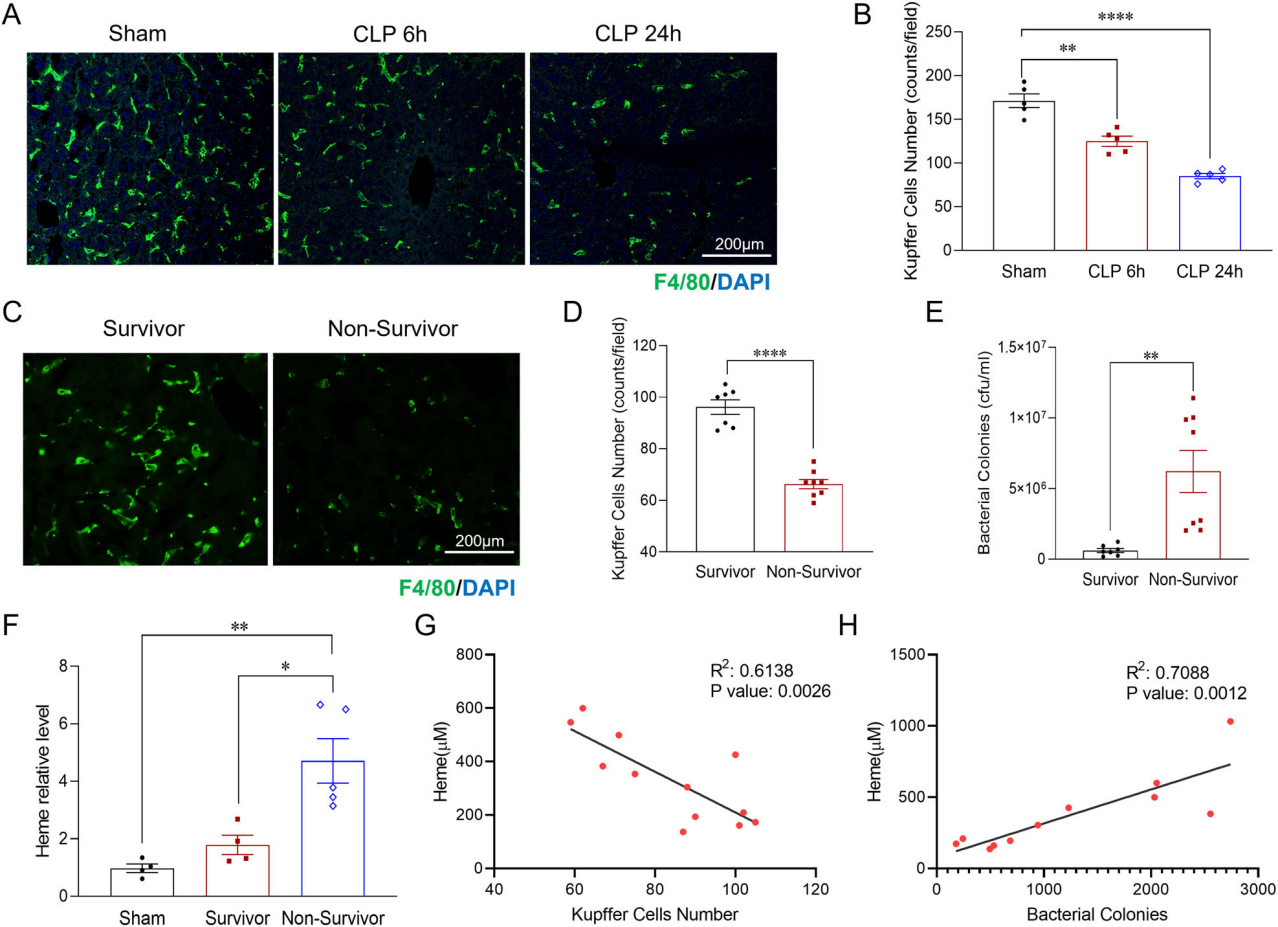
Sepsis-induced Kupffer cell loss, associated with elevated heme levels, correlates with increased bacterial load and higher mortality. **A** Representative immunofluorescence staining of F4/80 (green), a Kupffer cell marker, in liver sections from the sham and septic mice 6 and 24 h post-CLP (*n* = 6 mice/group). Scale bars: 200 µm. **B** Quantification of Kupffer cell counts in sham and septic groups (*n* = 5 mice/group). **C** Immunofluorescence staining of F4/80 (green) in liver sections of septic survivors (body temperature >30 °C) and non-survivors (body temperature <30 °C) at 24 h post-CLP (*n* = 8 mice/group). Scale bars: 200 µm. **D** Quantification of Kupffer cell counts in the septic survivors and non-survivors (*n* = 8 mice/group). **E** Quantification of bacterial load in the indicated groups (*n* = 8 mice/group). **F** Plasma levels of circulating free Heme in sham, survivors, and non-survivors at 24 h post-CLP (*n* = 4–6 mice/group). **G**, **H** Correlation analysis of free Heme levels with Kupffer cell counts and bacterial load. (*n* = 12 mice/group). Data are presented as mean ± SD. Statistical significance: *p < 0.05, **p < 0.01, ****p < 0.0001. CLP cecal ligation and puncture.

Kupffer cells, the largest population of tissue macrophages, are essential for clearing invading pathogens during sepsis [28–30]. To examine the impact of sepsis on Kupffer cells, we utilized the cecal ligation and puncture (CLP) in C57BL/6 mice, a well-established model for studying polymicrobial sepsis [31, 32]. Liver tissues and plasma were collected at 6 and 24 h post-CLP. Immunostaining for the Kupffer cell marker F4/80 revealed a rapid decrease in Kupffer cell numbers by 27% as early as 6 h post-CLP, with a more pronounced reduction of 50% by 24 h compared to sham controls (Fig. 1A, B). These findings indicate severe Kupffer cell loss in response to septic challenge, potentially compromising the liver’s ability to control systemic infections. Next, we examined whether Kupffer cell loss correlates with increased bacterial load and mortality during sepsis. Since a body temperature below 30 °C is a reliable indicator of mortality in sepsis [33], we analyzed Kupffer cell numbers and bacterial load in survivors (body temperature >30 °C) and non-survivors (body temperature <30 °C) at 24 h post-CLP. The results revealed a significant reduction in Kupffer cell numbers accompanied by an increased bacterial load in non-survivors compared to survivors (Fig. 1C–E). These findings highlight the correlation between Kupffer cell loss, increased bacterial load and sepsis severity, emphasizing the critical role of Kupffer cells in bacterial clearance and survival during sepsis.

### Elevated heme levels are associated with Kupffer cell loss, increased bacterial load, and higher mortality in sepsis

Hemolysis and the subsequent release of free heme are common complications of sepsis [21, 34]. To assess the relationship between elevated free heme levels and sepsis severity, we measured circulating heme levels in survivors and non-survivors. As shown in Fig. 1F, non-survivors exhibited a more than a 3.8 fold increase in free heme levels compared to survivors, indicating a strong association between elevated heme levels and poor outcomes in sepsis. Given prior in vitro studies suggesting that heme contributes to PAMPs induced macrophage PANoptosis [14, 22], we further explored the correlation between heme levels, Kupffer cell death, and bacterial load in septic mice. Our analysis demonstrates that septic mice with higher heme levels exhibit a marked reduction in Kupffer cell density and a significant increase in bacterial load compared to those with lower heme levels (Fig. 1G, H). These findings indicate a positive correlation between circulating free heme levels, the severity of Kupffer cell loss and bacterial burden, suggesting that elevated heme plays a critical role in driving sepsis associated Kupffer cell death and compromised bacterial clearance.

### Heme administration worsens sepsis-induced Kupffer cell loss and senescence, impairs bacterial clearance, and increases mortality

**Fig. 2:**
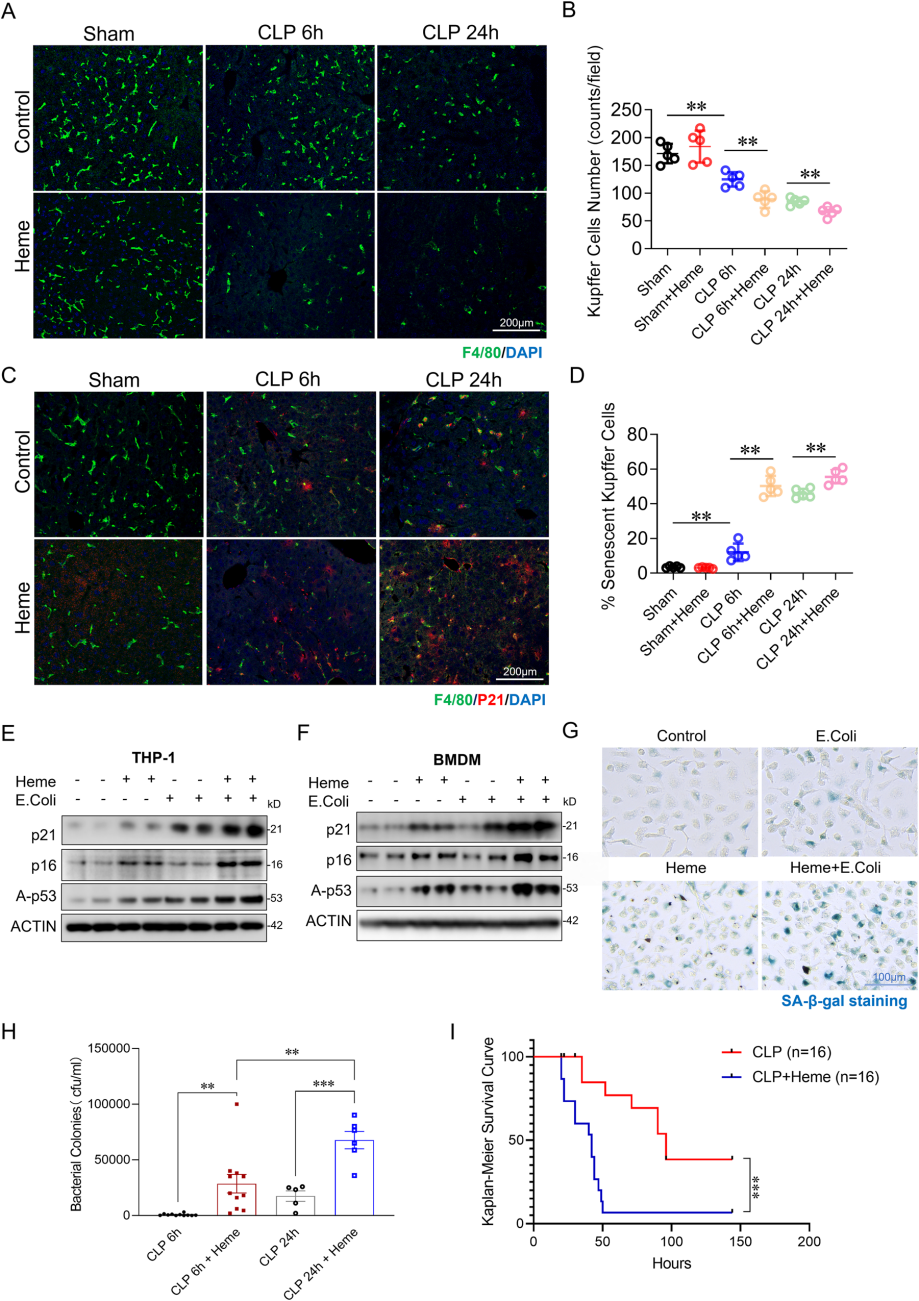
Heme administration exacerbates sepsis-induced Kupffer cell loss and senescence, impairs bacterial clearance, and increases mortality. **A** Representative immunostaining for F4/80 (green) in liver sections from sham, CLP-induced sepsis (6 and 24 h post-CLP), Heme + sham, Heme + CLP 6 h, and Heme + CLP 24 h groups (*n* = 5 mice/group). Scale bars: 200 µm. **B** Quantification of Kupffer cell counts in the indicated groups (*n* = 5 mice/group). **C** Immunofluorescence staining of p21 (red) and F4/80 (green) in the liver sections from same groups (*n* = 5 mice/group). Scale bars: 200 µm. **D** Quantification of p21 positive Kupffer cell counts in the indicated groups (*n* = 6 mice/group). Western blot analysis of senescence markers (p16, p21, and A-p53) in THP-1 cells (**E**) and BMDMs (**F**) treated with control, Heme (10 µM), heat-killed *E. coli* (MOI:10), or Heme + *E. coli* for 6 h. β-actin was used as a loading control. **G** SA-β-Gal staining in BMDMs treated with control, Heme, *E. coli*, or Heme + *E. coli* for 24 h (*n* = 6/group) Scale bars: 100 µm. **H** Bacterial colony counts in blood samples collected 6 and 24 h post-CLP in Heme-treated septic mice compared to untreated CLP controls. **I** Kaplan–Meier survival analysis of CLP and Heme + CLP groups (*n* = 16 mice/group). Each western blot represents 4 independent experiments. Data are presented as mean ± SD. Statistical significance: *p < 0.05, **p < 0.01, ***p < 0.001. THP-1: human monocytic cell line derived from an acute monocytic leukemia patient, BMDMs: bone marrow-derived macrophages, p21: p21^Cip1/Waf1, a cyclin-dependent kinase inhibitor that plays a critical role in cell cycle regulation, p16: p16^INK4a, a tumor suppressor protein that inhibits cyclin-dependent kinases 4 and 6 (CDK4/6), A-p53: Acetylated p53, a modified form of p53 that enhances its stability and transcriptional activity, SA-β-Gal: Senescence-associated β-galactosidase.

To further investigate the role of elevated heme in Kupffer cell loss, we administered exogenous heme (15 mg/kg, IV injection) or PBS to mice immediately following CLP-induced sepsis. Heme treated mice exhibited a significant reduction in Kupffer cell numbers at 24 h post-CLP sepsis compared to vehicle treated controls (Fig. 2A, B). Cellular senescence, characterized by cell cycle arrest and SASP factors secretion, impairs cell proliferation and self-renewal [35–37]. This dysfunction may impair Kupffer cell repopulation after severe depletion during sepsis, thereby compromising their ability to combat infection. To determine whether Kupffer cell senescence increases during sepsis, liver sections were co-stained for p21 (senescence marker) and F4/80. As shown in Fig. 2C, D, sepsis increased Kupffer cell senescence, which was further exacerbated in heme treated septic mice. Analysis of senescence markers p21, p16, and Acety-p53 in THP-1 cells and BMDMs exposed to heme, *E. coli*, or both confirmed that the combination markedly elevated senescence markers expression (Fig. 2E, F). Additionally, senescence-associated β-galactosidase (SA-β-Gal) staining further confirmed heightened levels of cellular senescence in the heme and *E.coli* co-treatment group (Fig. 2G). Notably, increased Kupffer cell loss and senescence correlated with higher bacterial loads and increased mortality in heme treated mice (Fig. 2H, I). These results suggest that elevated heme contributes to Kupffer cell death and senescence, impairing bacterial clearance and worsening sepsis outcomes.

### Heme exacerbates bacterial induced PANoptosis in macrophages

**Fig. 3:**
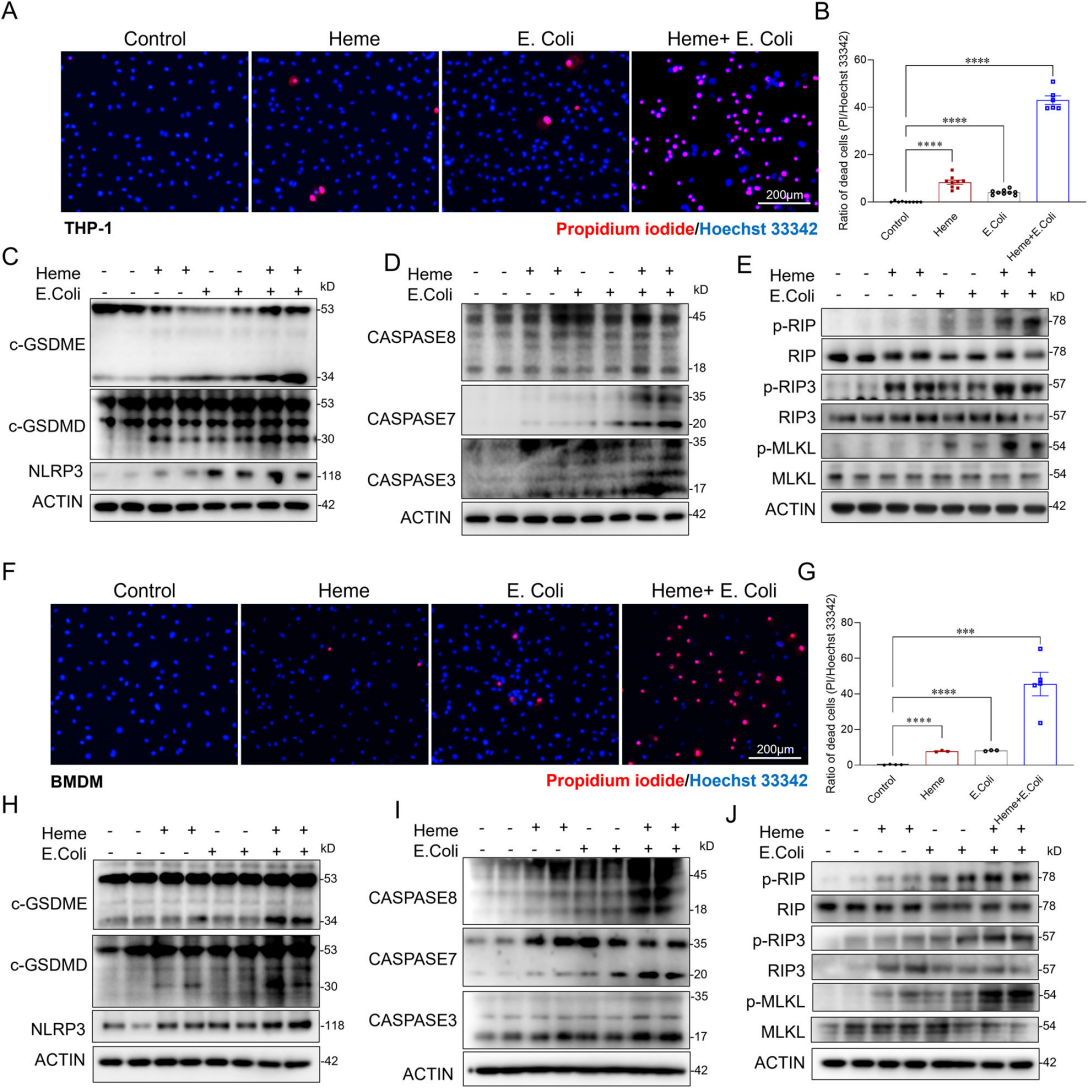
Heme exacerbates bacterial-induced PANoptosis in macrophages. **A** PI staining of THP-1 cells treated with control, Heme (10 µM), heat-killed *E. coli* (MOI:10), or Heme + *E. coli* for 24 h (*n* = 6–8/group) Scale bars: 200 µm. **B** Quantitative analysis of cell death (PI/Hoechst 33342) in THP-1 cells across the indicated groups (*n* = 6–8/group). **C**–**E** Western blot analysis of cell death pathways in THP-1 cells treated with control, Heme, heat-killed *E. coli*, or Heme + *E. coli* for 6 h. **C** Pyroptosis markers: NLRP3, c-GSDMD, c-GSDME; **D** Apoptosis markers: caspase-3, caspase-7, caspase-8; and **E** Necroptosis markers: phosphorylated MLKL, RIP, and RIP3. β-actin was used as a loading control. **F** PI staining of BMDMs treated under the same conditions (*n* = 4/group). Scale bars: 200 µm. **G** Quantitative analysis of cell death (PI/Hoechst 33342) of BMDMs in the indicated groups (*n* = 4/group). **H** Western blot analysis of pyroptosis markers: NLRP3, c-GSDMD, c-GSDME; **I** apoptosis markers: caspase-3, caspase-7, caspase-8; and **J** necroptosis markers: phosphorylated MLKL, RIP, and RIP3 in BMDMs treated with control, Heme, heat-killed *E. coli*, or Heme + *E. coli* for 6 h. β-actin was used as a loading control. Data are presented as mean ± SD. ***p < 0.001, ****p < 0.0001. PI propidium iodide, NLRP3 NOD-like receptor family pyrin domain-containing 3, c-GSDMD cleaved gasdermin D, c-GSDME cleaved gasdermin E, MLKL mixed lineage kinase domain-like protein, RIP receptor-interacting protein, RIP3 receptor-interacting protein kinase 3.

Our earlier findings indicate that elevated heme contributes to sepsis associated Kupffer cell death. To mimic the in vivo conditions of bacterial infection with elevated heme levels, we treated both BMDMs and THP-1 cells with heme (10 µM), heat killed bacterial (*E. coli*, MOI:10), or a combination of heme and E. coli for 6 and 24 h. Notably, treatment with heme or *E.coli* alone induced only slight cell death in both BMDMs and THP-1 cells, as revealed by propidium iodide (PI) staining. Specifically, heme treatment caused 7% cell death in BMDMs and 8% in THP-1 cells, while *E.coli* treatment resulted in 8% cell death in BMDMs and 4% in THP-1 cells. Strikingly, combined treatment with heme and *E.coli* dramatically increased cell death, reaching 45% in BMDMs and 43% in THP-1 cells (Fig. 3A, B, E, F). To investigate the mechanisms underlying this synergistic effect, we analyzed the activation of cell death related signaling pathways in response to heme plus *E.coli*, as well as heme or *E.coli* alone. As shown in Fig. 3C–E, H–J, treatment with heme or *E.coli* alone only minimally increased the activation of NLRP3/GSDMD/GSDME (pyroptosis), caspase-3/caspase7/caspase 8 (apoptosis), and pMLKL/pRIP3 (necrosis). In contrast, the combination of heme and *E. coli* significantly enhanced the activation of all three pathways in both THP-1 cells and BMDMs compared to either treatment with heme or *E.coli* alone (Fig. 3C–E, H–J). Collectively, these findings suggest that heme and bacterial stimuli synergistically induce severe cell death through PANoptosis—a coordinated cell death process involving pyroptosis, apoptosis, and necroptosis—rather than selectively activating a single pathway.

### Heme exacerbates bacterial induced mitochondrial damage

**Fig. 4:**
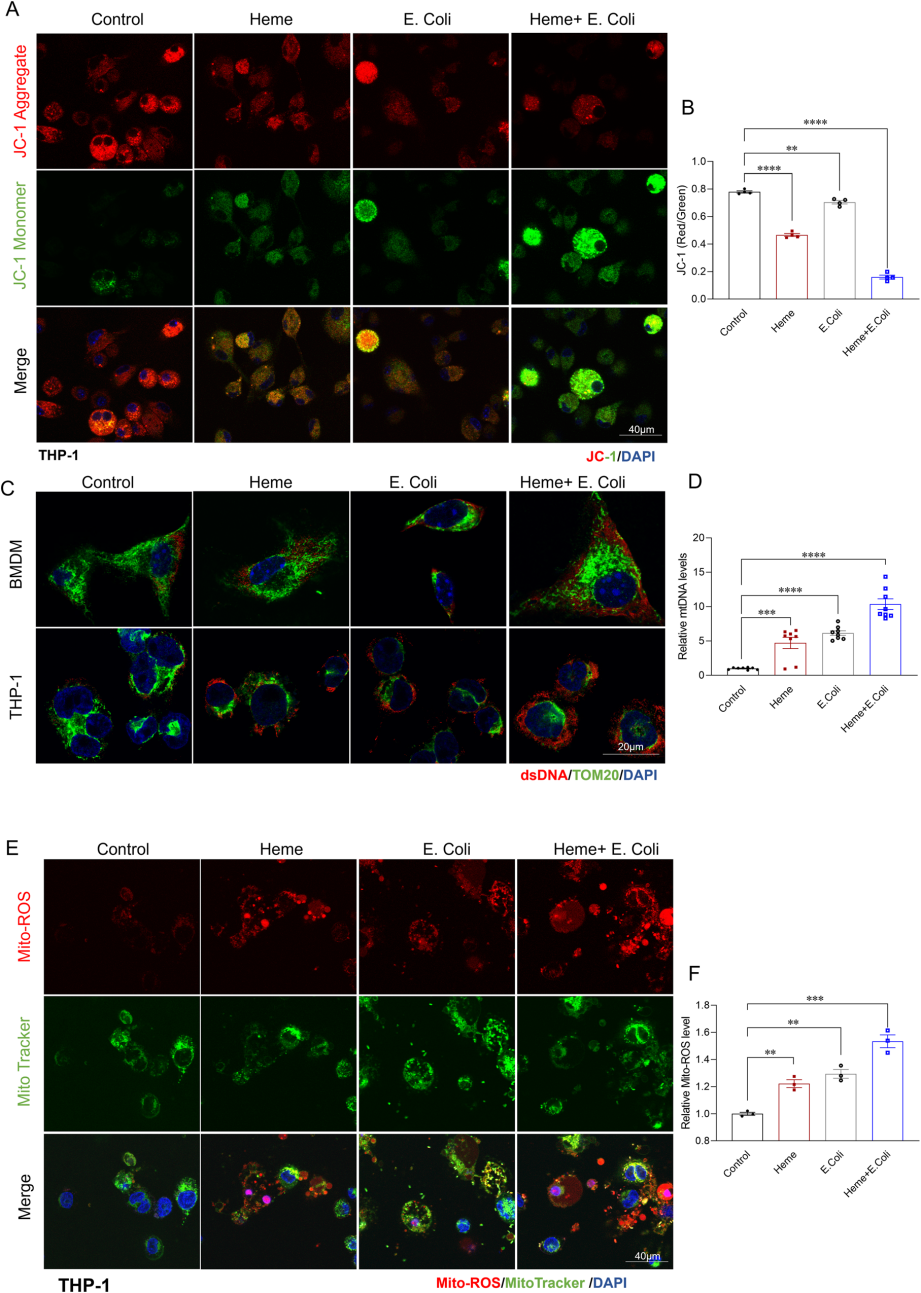
Heme exacerbates bacterial-induced mitochondrial damage in macrophages. **A** Representative JC-1 staining of mitochondrial membrane potential in live-THP-1 cells treated with control, Heme, *E. coli*, or Heme + *E. coli* for 3 h. (*n* = 4/group) Scale bars: 40 µm. **B** Quantitative analysis of JC-1 red/green fluorescence ratio in THP-1 cells across the indicated groups (*n* = 4/group). **C** Confocal images of BMDMs and THP-1 cells co-stained for dsDNA (red) and TOM20 (green) in the indicated groups (*n* = 6/group). Scale bars: 20 µm. **D** qPCR analysis of released Mito-DNA of BMDMs in the indicated groups (*n* = 8/group). **E** Representative images of THP-1 cells co-stained for Mito-ROS (red) and MitoTracker (green), after treatment with control, Heme, *E. coli*, or Heme + *E. coli* for 3 h. (*n* = 3/group) Scale bars: 40 µm. **F** Flow cytometry quantification of Mito-ROS in THP-1 cells treated as indicated (*n* = 3/group). Data are presented as the mean ± SD. **p < 0.01, ***p < 0.001, ****p < 0.0001. dsDNA double-stranded DNA, TOM20 translocase of the outer membrane 20, Mito-ROS mitochondrial reactive oxygen species.

Given the critical role of mitochondrial damage in triggering pyroptosis, apoptosis, necrosis, and cellular senescence [38–40], we sought to determine whether heme exacerbates mitochondrial damage during bacterial challenge. Both BMDMs and THP-1 cells were treated with either Heme, *E.coli* or both to mimic the conditions of infection in a high-heme environment. Mitochondrial integrity was evaluated by assessing three key indicators of mitochondrial health: mitochondrial membrane potential (JC-1 staining; Fig. 4A, B), mitochondrial DNA (mtDNA) release (Fig. 4C, D), and mitochondrial reactive oxygen species (ROS) production (Fig. 4E, F). As shown in Fig. 4A–F, treatment with either Heme or *E.coli* alone caused a slight increase in mitochondrial dysfunction, with membrane potential increasing by 40% and 9%, respectively (Fig. 4B), mtDNA release increasing by 37% and 51% (Fig. 4C, D), and ROS production increasing by 22% and 29% (Fig. 4E, F) in THP1 cells. However, the combination of heme and *E.coli* resulted in a significant increase in mitochondrial damage, with a 3.9 fold increase in mitochondrial membrane potential, a 10.4 fold increase in mtDNA release, and a 1.5 fold increase in mitochondrial ROS production. These findings suggest that heme exacerbates bacterial induced mitochondrial damage in macrophages, which may in turn drive the observed PANoptosis and cellular senescence during sepsis.

### PLC-γ mediated GSDMD mitochondrial translocation contributes to combined heme and bacterial induced mitochondrial damage

**Fig. 5:**
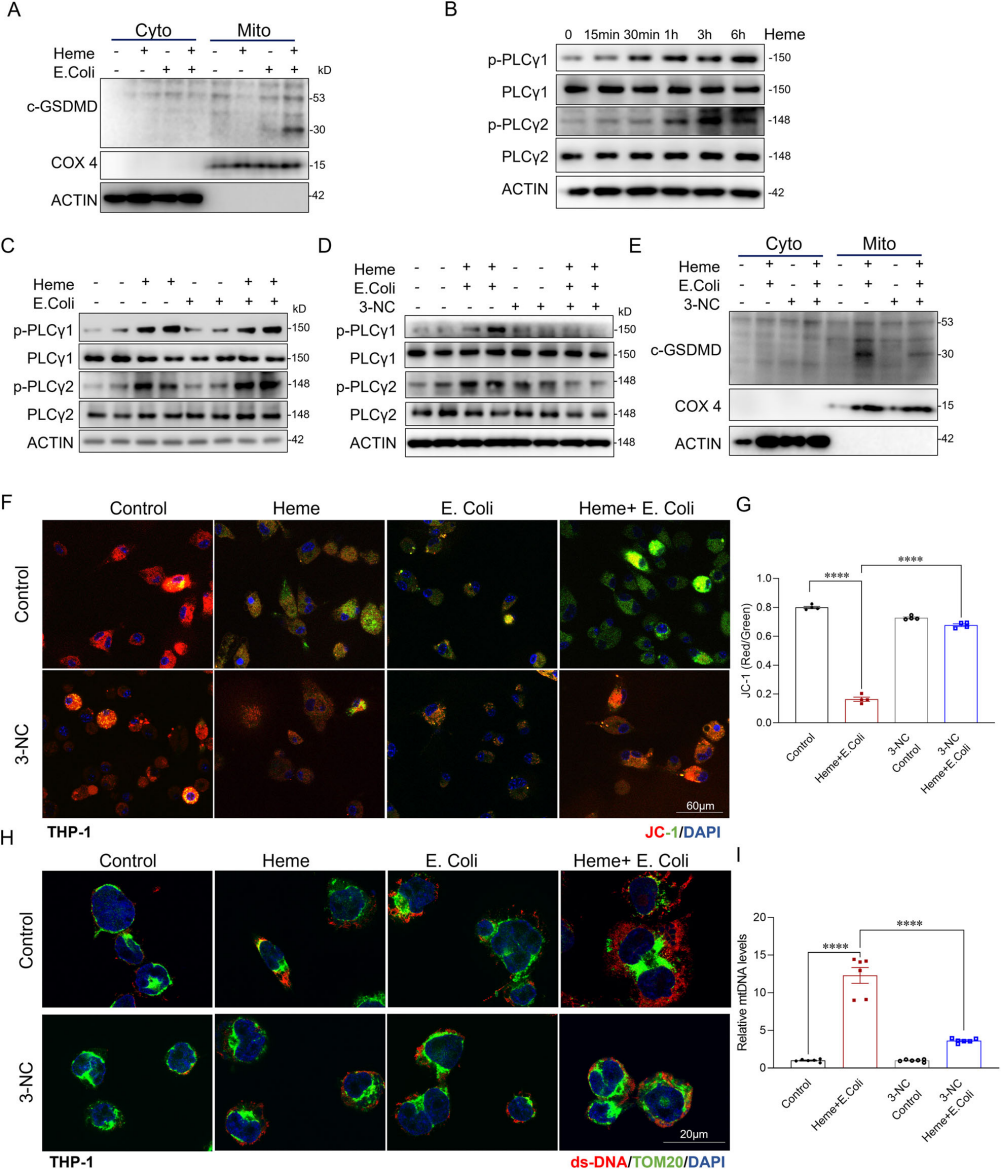
PLC-γ-mediated GSDMD mitochondrial translocation contributes to combined Heme and bacterial-induced mitochondrial damage. **A** Western blot analysis of cytosolic and mitochondrial fractions from THP-1 cells treated with control, Heme, *E. coli*, or Heme + *E. coli* for 6 h, showing c-GSDMD levels in the indicated groups. β-actin and COX4 were used as loading controls for cytosolic and mitochondrial fractions, respectively. **B** The protein expression of p-PLCγ1, PLCγ1, p-PLCγ2 and PLCγ2 was detected by western blot in THP-1 cells treated with heme at various time points (0 min, 15 min, 30 min, 1 h, 3 h, 6 h). β-actin was used as a loading control. **C** The protein expression of p-PLCγ1, PLCγ1, p-PLCγ2 and PLCγ2 was detected by western blot assay in THP-1 cells treated with control, Heme, *E. coli*, or Heme + *E. coli*. β-actin was used as a loading control. **D** Western blot analysis of p-PLCγ1, PLCγ1, p-PLCγ2 and PLCγ2 in THP-1 cells treated with control, Heme + *E. coli*, 3-NC + control, or 3-NC + Heme + *E. coli* for 6 h. β-actin was used as a loading control. **E** Western blot analysis of cytosolic and mitochondrial fractions of cleaved GSDMD from THP-1 cells in the indicated groups. β-actin and COX4 were used as loading controls for the cytosolic and mitochondrial fractions, respectively. **F** JC-1 staining in live-THP-1 cells treated with control, Heme, *E. coli*, Heme + *E. coli*, or 3-NC + Heme + *E. coli*. (*n* = 4/group). Scale bars: 60 µm. **G** Quantification of the red-to-green fluorescence ratio from JC-1 staining (*n* = 4/group). **H** Confocal images of THP-1 cells co-stained for ds-DNA (red) and TOM20 (green) in the indicated groups (*n* = 6/group). Scale bars: 20 µm. **I** qPCR analysis of released Mito-DNA of BMDMs in indicated groups (*n* = 6/group). Data are presented as mean ± SD. ***p < 0.001, ****p < 0.0001. 3-NC 3-Nitrocoumarin, c-GSDMD cleaved gasdermin D, JC-1 a dye used to assess mitochondrial membrane potential, with red fluorescence indicating healthy mitochondria and green fluorescence indicating depolarized or damaged mitochondria.

To investigate the mechanisms underlying mitochondrial damage following combined heme and bacterial exposure, we examined GSDMD cleavage and its subsequent mitochondrial translocation. Previous studies have shown that cGSDMD forms pores in mitochondrial membranes, leading to mitochondrial damage [41, 42]. To assess this, THP-1 cells were treated with heme, *E.coli*, or their combination. Cytosolic and mitochondrial fractions were purified. As shown in Fig. 5A, cGSDMD was undetectable in mitochondrial fractions following treatment with heme or *E.coli* alone. However, robust translocation of cGSDMD to mitochondria was observed upon combined exposure to heme and *E. coli*. These findings suggest that heme is critical in promoting cGSDMD translocation to mitochondrial.

To further explore the mechanistic link heme and cGSDMD mitochondrial translocation, we examined PLC-γ activation, which has been reported to contribute to GSDMD mediated pyroptosis [43]. As shown in Fig. 5B, heme treatment significantly increased PLC-γ activation, as indicated by increased phosphorylation levels. Further analysis demonstrated that heme significantly increased the phosphorylation of PLC-γ1 and PLC-γ2, whereas *E.coli* alone showed minimal activation (Figs. 5C and S1A). Next, we assessed whether PLC-γ inhibition reduces heme and bacterial induced GSDMD mitochondrial translocation and the resulting mitochondrial damage. THP-1 cells were pretreated with the PLC-γ inhibitor 3-Nitrocoumarin and then exposed to a combination of heme and bacterial. As shown in Fig. 5E, PLC-γ inhibition reduced cGSDMD translocation to mitochondria (Figs. 5E and S2A). Furthermore, PLC-γ inhibition alleviated mitochondrial damage in both THP-1 cells and human primary Kupffer cells, as evidenced by improved mitochondrial membrane potential (Figs. 5F, G and S2B), reduced mitochondrial DNA (mtDNA) release (Fig. 5H, I), and decreased mitochondrial ROS production (Fig. S3A) compared to the vehicle control. Western blot analysis confirmed that 3-NC effectively reduced PLC-γ activation in both THP-1 cells and BMDMs (Figs. 5D and S1B). These findings suggest that heme induced PLC-γ activation is a key driver of cGSDMD mitochondrial translocation and the resulting mitochondrial damage during combined heme and bacterial infection.

### PLC-γ inhibition mitigates heme- and bacterial-induced macrophage PANoptosis

**Fig. 6:**
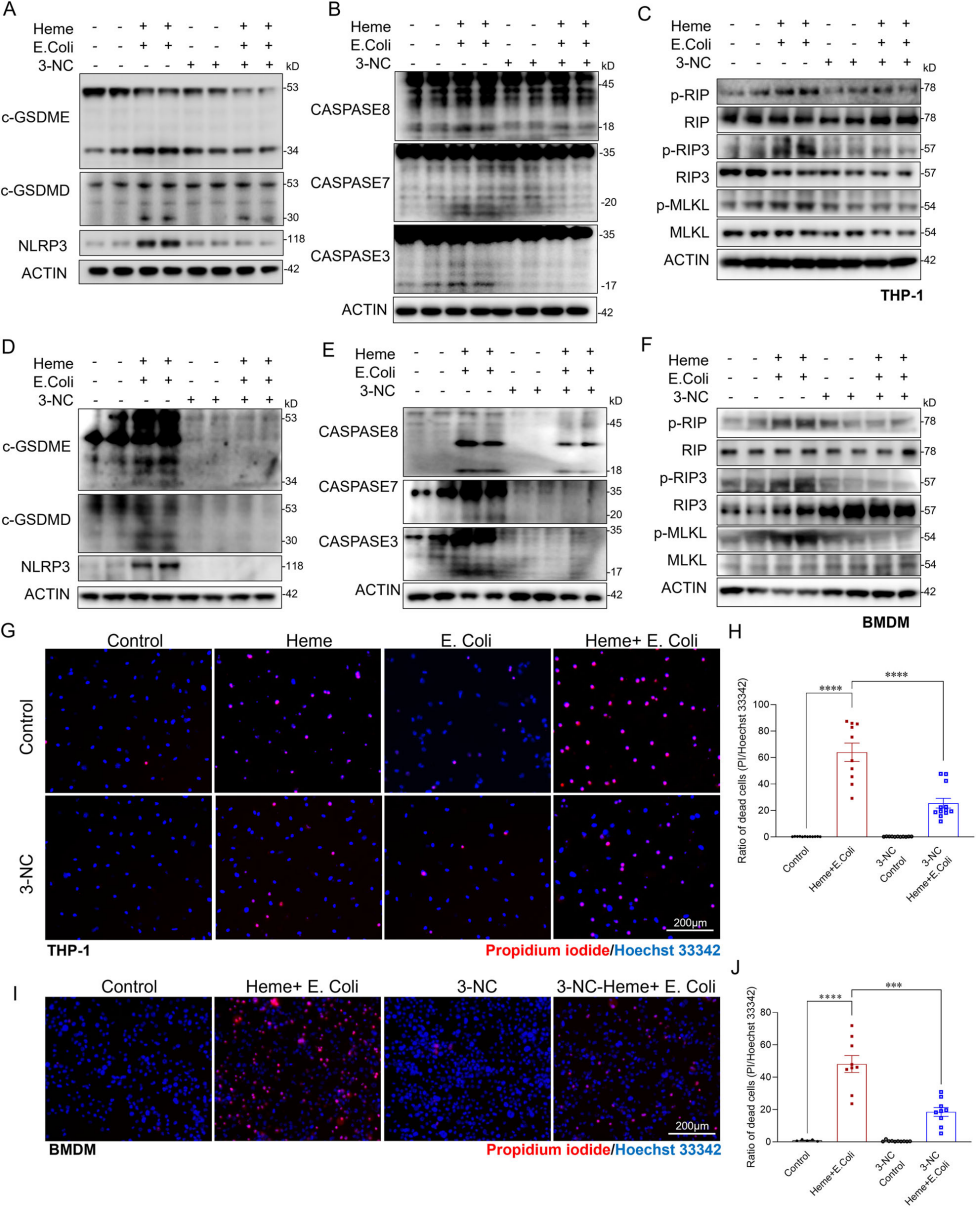
PLC-γ inhibition mitigates combined Heme and bacterial-induced PANoptosis in macrophages. **A**–**C** Western blot analysis of cell death pathways in THP-1 cells pre-treated with the PLC-γ inhibitor 3-NC (10 µM) or vehicle control, followed by exposure to Heme + *E. coli* for 6 h. **A** pyroptosis: NLRP3, c-GSDMD, c-GSDME; **B** apoptosis: caspase-3, caspase-7, caspase-8; and **C** necroptosis markers: phosphorylated MLKL, RIP, and RIP3. Western blot analysis of the same markers in BMDMs treated under identical conditions: **D** Pyroptosis markers: NLRP3, c-GSDMD, c-GSDME; **E** apoptosis: caspase-3, caspase-7, caspase-8; and **F** necroptosis markers: phosphorylated MLKL, RIP, and RIP3. β-actin was used as a loading control for all western blot analyses. **G** PI staining of THP-1 cells pre-treated with the PLC-γ inhibitor 3-NC (10 µM) or vehicle control, followed by exposure to Heme, *E. coli*, or Heme + *E. coli* for 24 h (*n* = 10/group) Scale bars: 200 µm. **H** Quantitative analysis of cell death (PI/Hoechst 33342) of THP-1 cells in the indicated groups (*n* = 10/group). **I** PI staining of BMDMs pre-treated with the PLC-γ inhibitor 3-NC (10 µM) or vehicle control, followed by treatment with Heme, *E. coli*, or Heme + *E. coli* for 24 h (*n* = 4–10/group) Scale bars: 200 µm. **J** Quantitative analysis of cell death (PI/Hoechst 33342) of BMDMs in the indicated groups (*n* = 4–10/group). Data are presented as mean ± SD. ***p < 0.001, ****p < 0.0001.

Given that PLC-γ inhibition reduced heme- and bacterial- induced cGSDMD mitochondrial translocation and mitochondrial damage, we next sought to determine whether targeting PLC-γ could alleviate macrophage death. THP-1 cells were pre-treated with 3-Nitrocoumarin before exposure to heme and *E.coli*. As shown in Fig. 6A–F, PLC-γ inhibition significantly reduced PANoptosis in macrophages, as evidenced by decreased cleavage and activation of GSDMD/GSDME and caspase-3/caspase 7, and reduced MLKL/RIP3 phosphorylation compared to controls. Additionally, PLC-γ inhibition reduced overall cell death in both THP-1 cells and BMDMs by approximately 60%, as indicated by propidium iodide (PI) staining (Fig. 6G–J). Given the role of heme-induced ROS production and ferroptosis, we investigated whether Ferrostatin-1, a synthetic antioxidant that prevents ferroptosis [44, 45], could attenuate cell death caused by heme and bacterial exposure. Treatment with Ferrostatin-1 significantly alleviated mitochondrial damage (Figs. S2C and S3) and reduced cell death (Fig. S4). Similarly, the use of a necrosis inhibitor also mitigated cell death induced by heme and *E.coli* (Fig. S4). These results suggest that PLC-γ is a critical mediator of heme- and bacteria-induced mitochondrial damage, cellular PANoptosis and ferroptosis, and that its inhibition may protect macrophages from the detrimental effects of combined heme and bacterial exposure during sepsis.

### cGAS-STING activation contributes to heme- and bacterial- induced macrophage senescence

**Fig. 7:**
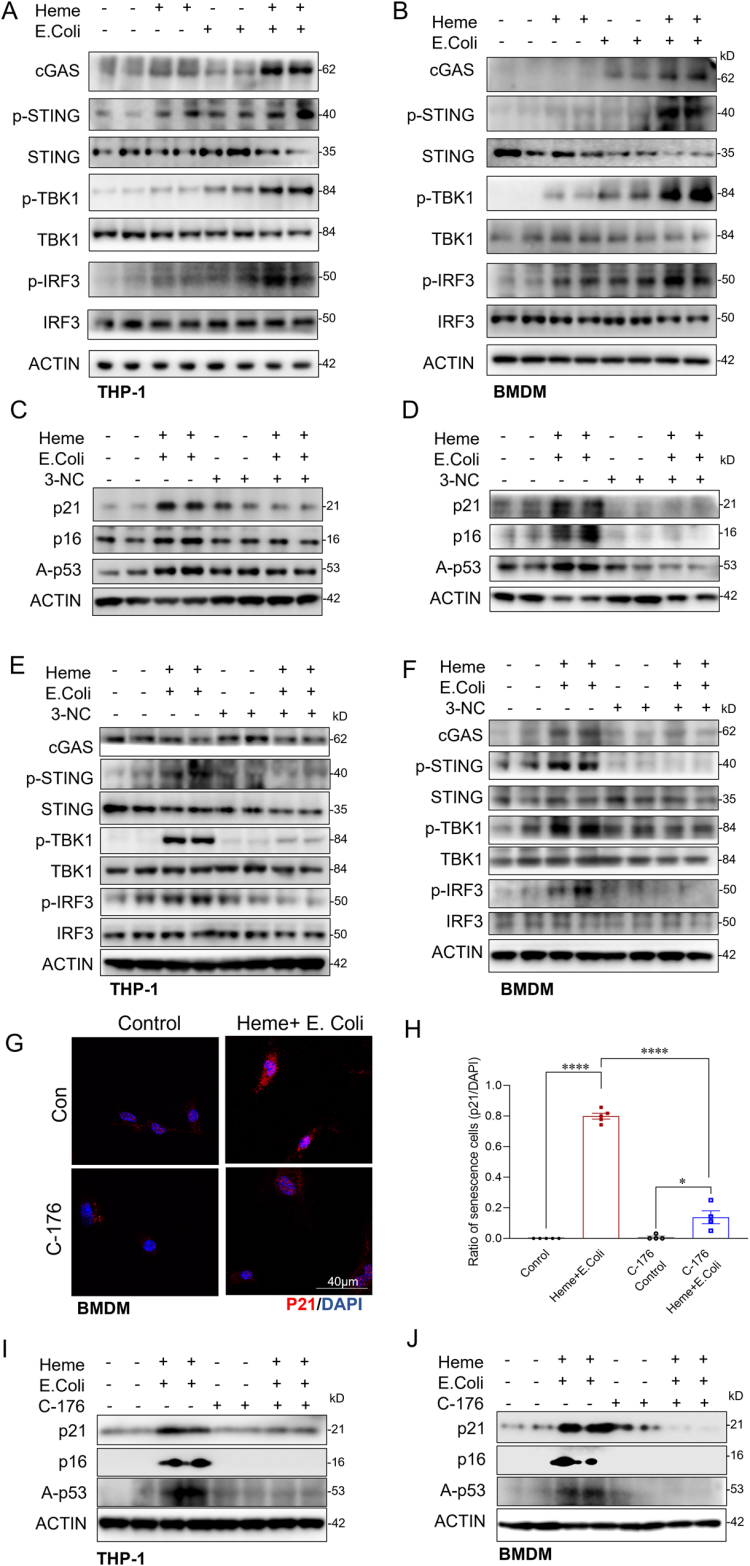
cGAS-STING activation contributes to macrophage senescence induced by combined Heme and bacterial exposure. **A**, **B** Western blot analysis of cGAS-STING pathway components (cGAS, p-STING, p-TBK1, p-IRF3) in THP-1 cells and BMDMs treated with control, Heme, *E. coli*, or Heme + *E. coli* for 6 h. **C**, **D** Western blot analysis of senescence markers (p21, p16, and A-p53) in THP-1 cells and BMDMs pre-treated with the PLC-γ inhibitor 3-NC (10 µM) or vehicle control, followed by exposure to Heme, *E. coli*, or Heme + *E. coli* for 6 h. **E**, **F** Western blot analysis of cGAS-STING pathway in THP-1 cells and BMDMs pre-treated with the PLC-γ inhibitor 3-NC (10 µM) or vehicle control, followed by exposure to Heme, *E. coli*, or Heme + *E. coli* for 6 h. β-actin was used as a loading control. **G** Confocal images of p21 in THP-1 cells treated with control or Heme + *E. coli*, with and without STING inhibitor (C-176) (*n* = 4–5/group) Scale bars: 40 µm. **H** Quantitative analysis of the ratio of p21 positive BMDMs in the indicated groups (*n* = 4–5/group). **I**, **J** Western blot analysis of senescence markers (p21, p16, and A-p53) in THP-1 cells and BMDMs pre-treated with C-176 (10 µM) or vehicle control, followed by exposure to Heme + *E. coli* for 6 h. β-actin was used as a loading control for all western blot analyses. Data are presented as mean ± SD. ****p < 0.0001. C-176 STING inhibitor, cGAS cyclic GMP-AMP synthase, p-STING phosphorylated STING (Stimulator of Interferon Genes), p-TBK1 phosphorylated TBK1 (TANK-binding kinase 1), p-IRF3 phosphorylated IRF3 (Interferon Regulatory Factor 3).

We next explored the mechanisms underlying macrophage senescence induced by heme and bacterial exposure, focusing on cGAS-STING activation by released mitochondrial DNA, a known senescence trigger [46, 47]. We first examined the effects of heme and bacterial exposure on cGAS-STING signaling activation in macrophages. Treatment with either heme or bacterial alone resulted in only a modest activation of the cGAS-STING signaling. However, combined exposure to heme and bacterial markedly enhanced activation of the cGAS-STING pathway, as evidenced by significantly increased phosphorylation of STING, TBK1, and IRF3 in THP-1 cells (Fig. 7A) and BMDMs (Fig. 7B).

To further investigate the role of cGAS-STING in macrophage senescence, we treated BMDMs with a STING inhibitor during heme-bacterial exposure. STING inhibition significantly reduced senescence markers, including p21 immunostaining (Fig. 7G, H) and the expression of p21, p16, and acetylated p53 (Fig. 7I, J). Moreover, PLC-γ inhibition, which alleviated heme and bacterial induced mitochondrial damage and mtDNA release (Fig. 5H–J), also reduced cGAS-STING pathway activation and cellular senescence (Fig. 7C–F). Collectively, these findings indicate that mitochondrial damage caused by combined heme and bacterial exposure leads to cGAS-STING activation, contributing to macrophage senescence.

### Increased hemopexin expression alleviates sepsis-induced Kupffer cell loss and improves survival in both young and aged mice

**Fig. 8:**
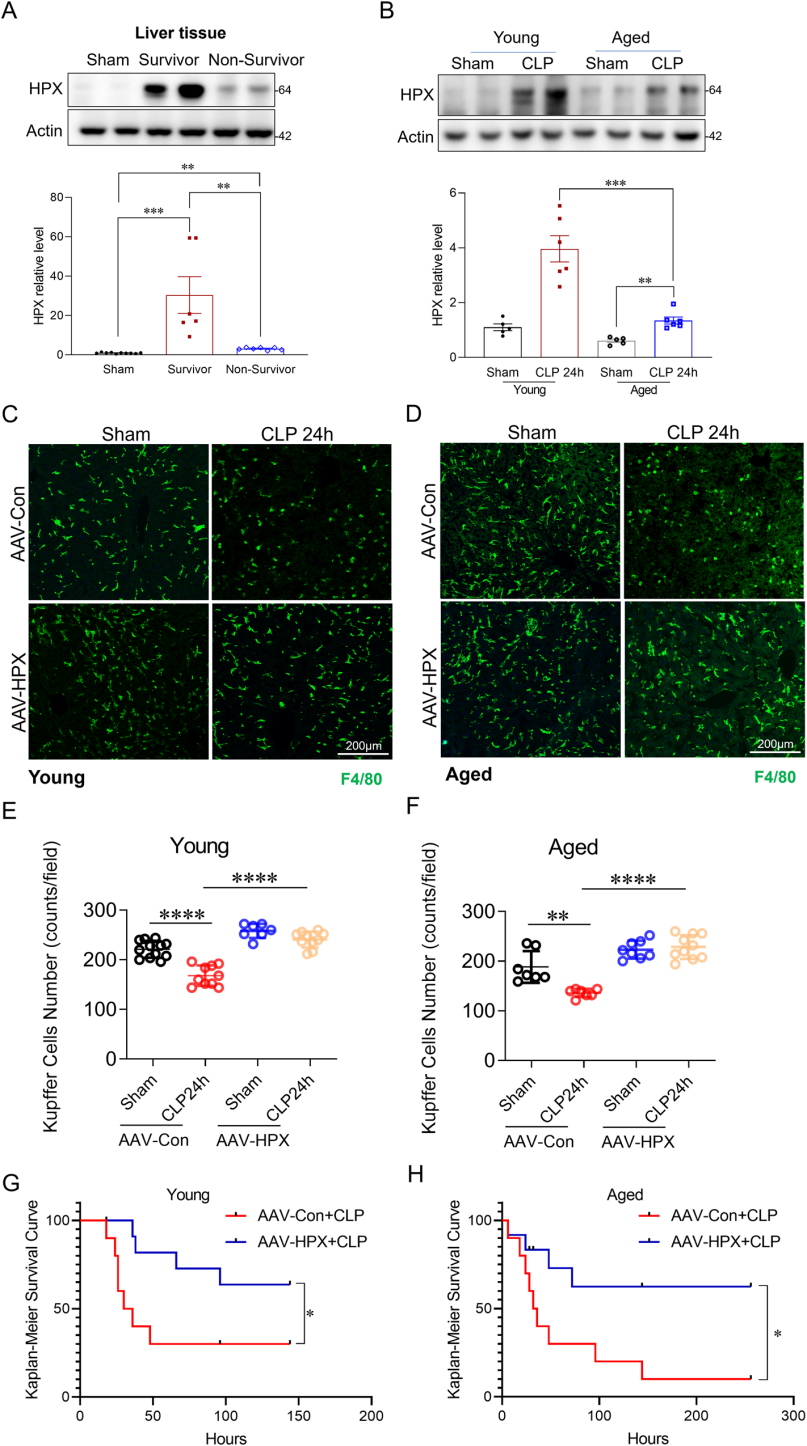
Increased hemopexin expression alleviates sepsis-induced Kupffer cell loss and improves survival in both young and aged mice. **A** Western blot analysis and quantification of HPX expression in liver tissues from sham, septic survivors, and septic non-survivors 24 h post-CLP (*n* = 6/group). **B** Western blot analysis and quantification of HPX expression in young and aged mice subjected to CLP-induced sepsis or sham treatment (*n* = 6–8/group). β-actin was used as a loading control for all western blot analyses. **C**, **D** Representative immunofluorescence staining of F4/80 (green) in liver sections from young and aged mice treated with AAV-HPX or AAV-Con and subjected to sepsis 24 h post-CLP (*n* = 6–8/group). Scale bars: 200 µm. **E**, **F** Quantification of Kupffer cell counts in young and aged mice across the indicated treatment groups (*n* = 6–8/group). **G** Kaplan–Meier survival analysis of young and aged mice pre-treated with AAV-HPX or AAV-Con (*n* = 10/group). **H** Comparison of survival rates between young and aged mice with increased HPX expression. Data are presented as mean ± SD. *p < 0.05, **p < 0.01, ***p < 0.001, ****p < 0.0001. HPX Hemopexin, AAV-HPX adeno-associated virus encoding HPX, AAV-Con adeno-associated virus control.

Hemopexin (HPX), a liver derived heme scavenger, plays a crucial role in clearing free heme from circulation [48–50]. Our previous studies indicate that circulating free heme levels are significantly higher in non-survivors than in survivors (Fig. 1F). Notably, HPX expression is upregulated during sepsis (Fig. 8A, B), and survivors exhibit higher HPX levels compared to non-survivors (Figs. 8A and S6A), suggesting a protective role of HPX in sepsis outcomes. Aging is a known risk factor for bacterial infections and progression to severe sepsis. We observed that aged mice exhibited an 85% increase in free heme levels (Fig. S5A), and a 3.3-fold higher bacterial load (Fig. S5B, C), accompanied by severe Kupffer cell loss (Fig. S5D, F), senescence (Fig. S5D, G), and increased mortality compared to young mice (Fig. S5E) during sepsis. Notably, aged mice also demonstrated a 66% reduction in HPX expression in response to sepsis compared to young mice (Figs. 8B and S6B). To assess whether increasing HPX expression could mitigate sepsis severity, we administered AAV-HPX, with AAV-Con as a control. HPX expression levels in liver tissues (Fig. S6C) and plasma (Fig. S6D) were assessed by Western blot and ELISA. Two weeks after AAV delivery, the mice were subjected to CLP induced sepsis. As shown in Fig. S6E, F, enhanced HPX expression significantly reduced circulating free heme levels and bacterial loads (Fig. S6G, H), alleviated sepsis induced Kupffer cell loss (Fig. 8C–F) and senescence (Fig. S7A–D), and improved survival (Fig. 8G, H) in both young and aged mice. These results demonstrate that increased HPX expression effectively mitigates sepsis severity by reducing free heme levels, enhancing bacterial clearance, and protecting against Kupffer cell death and senescence, thereby improving survival outcomes in both young and aged mice.

## Discussion

This study uncovers a previously unrecognized mechanism linking elevated free heme levels and Kupffer cell loss during sepsis. We demonstrate that heme exacerbates bacterial induced mitochondrial damage, leading to Kupffer cell death via PANoptosis and promoting immune senescence through cGAS-STING activation. These findings underscore the central role of mitochondrial dysfunction in sepsis induced immune impairment and highlight potential therapeutic targets for mitigating sepsis associated mortality, particularly in aging populations.

Kupffer cells are essential for pathogen clearance, immune homeostasis, and infection resolution [51–54]. Their depletion during sepsis is strongly associated with impaired bacterial clearance and increased mortality (Fig. 1). Previous studies have suggested that heme enhances PAMP-induced cell death via activation of the NLRP12 and NLRC5 mediated PANoptosome complex [14, 22]. However, our findings reveal a distinct mechanism, in which mitochondrial damage serves as the central driver of heme- and bacterial-induced Kupffer cell death. We show that heme and bacterial infection synergistically exacerbate mitochondrial damage, as evidenced by a significant loss of mitochondrial membrane potential, increased mitochondrial DNA release, and elevated mitochondrial ROS production. Given the critical role of mitochondrial damage in amplifying cell death pathways [55, 56], we further dissected the underlying mechanisms. Our data demonstrate that heme- and bacterial-induced mitochondrial damage triggers not only GSDMD-mediated pyroptosis but also activates the RIPK-MLKL mediated necroptosis and caspase-dependent apoptosis, resulting in a synergistic form of cell death known as PANoptosis [14, 22]. As Kupffer cells are the major site for heme clearance [57], their loss during sepsis may further exacerbate free heme accumulation, thereby driving a detrimental cycle of mitochondrial damage, cell death, and immune dysfunction. This maladaptive cascade may contribute to persistent immune dysfunction, impaired pathogen clearance, and worsened clinical outcomes in sepsis. Therefore, maintaining Kupffer cell viability is critical for mitigating heme-induced immune dysregulation and improving host resistance to infection.

Cellular senescence, characterized by irreversible cell cycle arrest and a pro-inflammatory senescence associated secretory phenotype (SASP), plays a pivotal role in immune aging and dysregulation [58–60]. Kupffer cell senescence compromises self-renew capacity and impairs repopulation following severe depletion during sepsis. While the role of sepsis in Kupffer cell senescence remains poorly understood, our findings indicate that sepsis accelerates Kupffer cell senescence, particularly in the presence of elevated heme levels. We identify heme as an amplifier of bacterial induced cGAS-STING activation, which serves as a key driver of heme induced Kupffer cell senescence. Notably, pharmacological inhibition of the cGAS-STING pathway attenuates Kupffer cell senescence, highlighting a potential therapeutic target for preventing immune aging in sepsis.

A key mechanistic finding of this study is the identification of PLC-γ as a central mediator of heme- and bacterial-induced mitochondrial dysfunction. We demonstrate that heme activates PLC-γ, which facilitates cGSDMD translocation to mitochondria, thereby promoting mitochondrial damage. Importantly, PLC-γ inhibition effectively reduces cGSDMD mitochondrial translocation, attenuating the activation of pyroptotic, apoptotic, necroptotic, and senescence associated pathways. These findings suggest that targeting PLC-γ could be a promising therapeutic strategy to mitigate Kupffer cell loss and improve sepsis outcomes. However, the precise molecular mechanisms by which PLC-γ regulates cGSDMD mitochondria translocation remain unclear and are worth further investigation.

Given the deleterious effects of free heme, we investigated the therapeutic potential of hemopexin (HPX), a heme scavenger [21]. We found that increased HPX expression provided significant protection against Kupffer cell death and senescence, leading to enhanced bacterial clearance and improved survival, particularly in aged mice. Notably, aging is associated with reduced HPX levels, which may contribute to increased heme medicated immune dysfunction. The findings suggest that HPX administration may serve as a promising strategy to mitigate sepsis severity.

In conclusion, this study provides a comprehensive mechanistic understanding of how elevated free heme drives mitochondrial damage, Kupffer cell death, and immune senescence during sepsis. By identifying PLC-γ and the cGAS-STING pathway as key mediators of these processes, and highlighting the potential of HPX administration, this research offers promising therapeutic strategies for addressing sepsis-associated immune dysfunction.

## Materials and methods

### Animals and housing

Wild-type C57BL/6 mice were obtained from Jackson Laboratory and housed at the Institutional Animal Care and Use Facility at East Tennessee State University. Male and female mice aged 3–5 months were categorized as young, while those aged 22–24 months were classified as aged. All animal care and experimental procedures were approved by the ETSU Committee on Animal Care and Use and adhered to NIH guidelines to ensure humane treatment.

### Sepsis model: cecal ligation and puncture (CLP)

Polymicrobial sepsis was induced using the cecal ligation and puncture (CLP) model as described previously. Briefly, mice were anesthetized with isoflurane and positioned on a heated surgical pad. Following a midline incision, approximately one-third of the cecum was ligated and punctured once using a 23-gauge needle. After repositioning the cecum, the abdominal cavity was closed. The peritoneal wall was sutured with sterile 4-0 Dafilon sutures and the skin was closed with a surgical staple. Sham-operated mice underwent similar procedures without ligation or puncture. Following CLP surgery, mice were administered a single subcutaneous dose of resuscitative fluid. Liver tissues and plasma were collected at 6 and 24 h post CLP for immunostaining, heme level quantification, and bacterial colony analysis.

### Supplementary methods and materials

Detailed methods for cell culture and treatment, AAV packaging and administration, immunostaining, heme level measurement, mitochondrial isolation, protein extraction, Western blot analysis, and a list of antibodies are provided in the Supplementary Materials.

### Statistical analysis

All experiments were performed at least three independent times and representative data are shown. Statistical analysis between two groups were performed by a two-tailed Student’s *t* test, while one-way ANOVA with Tukey’s post hoc test was used for multiple comparisons. A *p* value less than 0.05 was considered statistically significant. Data are expressed as mean ± SD. Analyses were performed using GraphPad Prism 8.4.3 software.

## Supplementary information

Supplementary data

Full and uncropped western blots

## Data Availability

All data generated or analyzed during this study are available from the corresponding author upon reasonable request.
