# Supplementary material for: The role of heme in sepsis induced Kupffer cell PANoptosis and senescence
Source: Cell Death Dis. 2025 Apr 13;16(1):284. doi: 10.1038/s41419-025-07637-6 (PMC11993645; doi:10.1038/s41419-025-07637-6)
Supplement: Supplementary file 1 — Supplementary data [file 41419_2025_7637_MOESM1_ESM.docx]

**The Role of Heme in Sepsis Induced Kupffer Cell PANoptosis and Senescence**

Authors: Tingting Li, Joseph Adams, Peilin Zhu, Tao Zhang, Fei Tu, Amy Gravitte, Xiaojin Zhang, Li Liu, Jared Casteel, Valentin Yakubenko, David L Williams, Chuanfu Li, Xiaohui Wang

**Table of contents**

Supplementary methods and Materials.....................................................................................2

Supplementary figures...............................................................................................................5

**Adeno-Associated Virus (AAV) packaging and in vivo Administration**

Recombinant adeno-associated virus (AAV) vectors expressing the human hemopexin (HPX) gene under a CMV promoter (pAAV-CMV-HPX, ABM, Cat: 23884101) were generated using a triple-plasmid system, including a packaging plasmid (pAAV2/8, Addgene Cat: 112864) and a helper plasmid (pAdDeltaF6, Addgene Cat: 112867). The pAAV-CMV-Blank vector served as a negative control. HEK293T cells were co-transfected with these plasmids using Lipofectamine 3000, and viral particles were harvested 72 hours later. HEK293T cells were co-transfected with the plasmids using Lipofectamine 3000, and viral particles were harvested 72 hours post-transfection. Viral purification and titering were performed according to published protocols. Mice received intravenous injections of AAV-HPX or control AAV at a single dose of 1 × 10¹¹ genome copies (GC) in 100 µL of sterile PBS via the tail vein. Two weeks post-AAV delivery, the cecal ligation and puncture (CLP) model of sepsis was performed as previously described. Transfection efficiency was confirmed by assessing HPX expression in liver tissues.

**Immunofluorescence, confocal imaging, and analysis**

At 6 and 24 hours post-CLP, both sham and septic mice were anesthetized, and liver tissues were collected and fixed in 4% paraformaldehyde (PFA) for 12 hours at 4°C, followed by immersion in 30% sucrose overnight. Tissues were embedded in OCT and sectioned into 10μm slices using a Leica CM3050S cryostat. Cryosections were washed three times with PBS, permeabilized for 20 min with 0.5% Triton X-100 in PBS at room temperature and then blocked with 10% goat serum for 1 hours. Sections were incubated with primary antibodies against F4/80 for Kupffer cells (Bio-Rad, Cat: Cl:A3-1, 1:100), and p21 for senescence (CST, Cat: 2947S, 1:100) at 4°C overnight. Following washing once with PBST and twice with PBS, sections were incubated with Alexa Fluor-conjugated secondary antibodies at room temperature for 1 hours. Following three times PBST washes, stained sections were mounted in fluorescence mounting medium with 4′,6-diamidino-2-phenylindole (DAPI) (ThermoFisher, Cat: P36981). Images were acquired using a Leica TCS SP8 confocal microscope and analyzed with ImageJ software (NIH).

**Colony-forming units**

Blood bacteria loads were quantitated as colony forming units (CFU) at 6 and 24 hours post CLP. Briefly, blood samples were collected from the mouse tail under anesthesia and diluted with sterile PBS. Aliquots were plated on Tryptic Soy Agar plates with 5% sheep blood (Hardy Diagnostics, Cat: A10) and incubated at 37°C for 16 hours. CFUs were counted to determine bacterial burden.

**Measurement of Heme Levels in plasma**

Blood samples were collected via cardiac puncture into tubes containing heparin and centrifuged at 3000g for 20 minutes to separate plasma. Plasma heme levels were measured using the Heme Assay Kit (Sigma-Aldrich, Cat:MAK316) according to the manufacturer’s instructions. Plasma hemopexin (HPX) levels were quantified using an ELISA kit (Abclonal, Cat: RK09237)

**Cell Culture**

Bone marrow-derived macrophages (BMDMs) were isolated from the femurs and tibias of C57BL/6 mice aged 3–5 months following established protocols. Briefly, mice were euthanized via CO₂ inhalation, and femurs and tibias were harvested aseptically. Bones were cleaned of soft tissue and placed in ice-cold PBS. Under sterile conditions, both ends of each bone were clipped, and bone marrow was flushed out using a 25-gauge needle attached to a syringe containing RPMI-1640 medium supplemented with 10% FBS and 1% penicillin-streptomycin. The marrow suspension was gently pipetted to dissociate clumps, filtered through a 70 μm cell strainer, and centrifuged at 500 × g for 5 minutes at 4°C. The cell pellet was resuspended in RPMI-1640 complete medium, and cell viability was confirmed using trypan blue exclusion. For macrophage differentiation, bone marrow cells were cultured in RPMI-1640 medium supplemented with 20 ng/mL recombinant mouse macrophage colony-stimulating factor (M-CSF; Thermo Fisher Scientific, Cat: PMC2044) at 37°C in a humidified atmosphere with 5% CO₂. On day 3, half the medium was replaced with fresh M-CSF-containing medium, and cultures were maintained for a total of 7 days to generate macrophages for downstream applications. THP-1 human monocyte-derived macrophages (ATCC, Cat: TIB-202 ™) were cultured in RPMI-1640 medium supplemented with 2 mM L-glutamine, 1% penicillin-streptomycin, and 10% FBS. Differentiation into macrophages was induced by treating cells with 100 nM phorbol 12-myristate 13-acetate (PMA) for three days. Cells were washed twice with pre-warmed PBS to remove residual PMA and rested for 24 hours before experimental use.

**Cell treatment and assays for cell death, senescence, and mitochondrial damage**

Bone marrow-derived macrophages (BMDMs) and THP-1 macrophages were treated with the following stimuli and inhibitors, alone or in combination, as indicated: heme (10 μM, Hemin, Sigma, Cat: 51280-5G), heat-killed E. coli (MOI: 20, ATCC, Cat: BAA197), PLC-γ inhibitor (10 μM, 3-NC, Cat: HY-111919), STING inhibitor (2.5µM, C-176, MCE, Cat: HY-112906). F-1 (10µM, Ferrostatin-1, MCE, Cat: HY-100579), MLKL inhibitor (10µM, Necrosulfonamide, MCE, Cat: HY-100573). Cells were treated for the indicated times to assess mitochondrial ROS production using MitoSOX Red (Invitrogen, Cat: M36008), and mitochondrial membrane potential using JC-1 staining (MCE, Cat: HY-15534). Cell death was evaluated using propidium iodide (PI) staining (MCE, Cat: HY-D0815). Mitochondrial ROS and membrane potential assessments, along with PI staining, were analyzed by flow cytometry (BD FACSymphony™ A3 Cell Analyzer), confocal imaging (TCS SP8), and a fluorescence plate reader (SpectraMax 340PC384), adhering to the respective manufacturer protocols. Cell senescence was determined via β-galactosidase (SA-β-Gal) activity using the Senescence β-Galactosidase Staining Kit (CST, Cat: 9860S). Additionally, the expression of senescence markers was analyzed by Western blot and immunostaining.

**Protein Extraction and Western Blot Analysis**

Protein extraction from liver tissues and in vitro cultured cells was performed using RIPA lysis buffer (Thermo Fisher Scientific, Cat: 89900) supplemented with protease and phosphatase inhibitors (Sigma-Aldrich, Cat: PPC2020). Protein concentrations were measured using a BCA protein assay kit (Thermo Fisher Scientific, Cat: 23223). Equal amounts of protein were separated by electrophoresis on Bis-Tris protein gels and transferred onto nitrocellulose membranes. Membranes were blocked with 5% BSA in TBS containing 0.5% Tween-20 (TBST) for 1 hour at room temperature, followed by incubation with specific primary antibodies overnight at 4°C. After washing with TBST, membranes were incubated with HRP-conjugated secondary antibodies (Cell Signaling Technology, Cat: 7074) for 1 hour at room temperature, followed by additional washes with TBST to remove unbound antibodies. Bands were visualized using the SuperSignal™ West Femto Maximum Sensitivity Substrate (Thermo Fisher Scientific, Cat: 34096), optimized for detecting low-abundance targets, and SuperSignal™ West Pico PLUS Chemiluminescent Substrate (Thermo Fisher Scientific, Cat: 34580) for higher-abundance targets. Imaging was conducted with the G:Box Chemi gel documentation system (GeneSys Version: 1.8.10.0), and band intensities were quantified using Image J software.

**Antibodies**

The following primary and secondary antibodies were used in this study: Anti-STING (Cat: 13647S), anti-p-STING (Cat: 72971S), anti-TBK1 (Cat: 3504S), anti-p-TBK1 (Cat: 5483S), anti-IRF3 (Cat: 4302S), anti-p-IRF3 (Cat: 4947S), anti-p-IRF3 (Cat: 4947S), anti-p-IRF3 (Cat: 4947S), anti-p-IRF3 (Cat: 4947S), anti-cGAS (Cat: 31659S), anti-NLRP3 (Cat: 15101S), anti- Gasdermin E (Cat: 88874S), p21 (Cat: 2947S) were purchased from Cell Signaling Technology. The Necroptosis antibody sampler kit (Cat: 98110T) which contains Anti-p-RIP, Anti-RIP, Anti-p-RIP3, Anti-RIP3, Anti-p-MLKL and Anti-MLKL, was purchased from Cell Signaling Technology. Anti-c- Gasdermin E (Cat: A26197), Anti-c- Gasdermin D (Cat: A24059), Anti-P16(Cat:A8571), Anti-AP53 (Cat:A19836), Anti- Phospho-PLC gamma 1 (Cat:AP1340), Anti- PLC gamma 1 (Cat:A8899), Anti- Phospho-PLC gamma 2 (Cat:AP1519), Anti- PLC gamma 2 (Cat:A5182), Anti- Hemopexin (HPX) (Cat:A5603) were purchased from Abclonal. Anti-Caspase-8(Cat: 13423-1-AP), Anti-Caspase-7(Cat: 27155-1-AP), Anti-Caspase-3(Cat: 19677-1-AP), Anti-TOM20(Cat: 66777-1-Ig) were purchased from Proteintech.

**Mitochondrial isolation and mtDNA Release Assay**

Mitochondria were isolated using Cell Mitochondria Isolation Kit (Thermo Fisher Scientific, Cat: 89874) according to manufacturer’s instructions. DNA was extracted from fractionated cytosolic fractions or isolated mitochondria using the DNeasy Blood & Tissue Kit (Qiagen, Cat: 69504) following the manufacturer’s instructions. Mitochondrial DNA (mtDNA) levels were quantified by qRT-PCR using specific primers for Cytochrome c oxidase I (forward: 5’-GCCCCAGATATAGCATTCCC-3’, reverse: 5’-GTTCATCCTGTTCCTGCTCC-3’). Results were normalized to 18S rRNA levels (forward: 5’-TAGAGGGACAAGTGGCGTTC-3’, reverse: 5’-CGCTGAGCCAGTCAGTGT-3’) to account for variation in sample input.


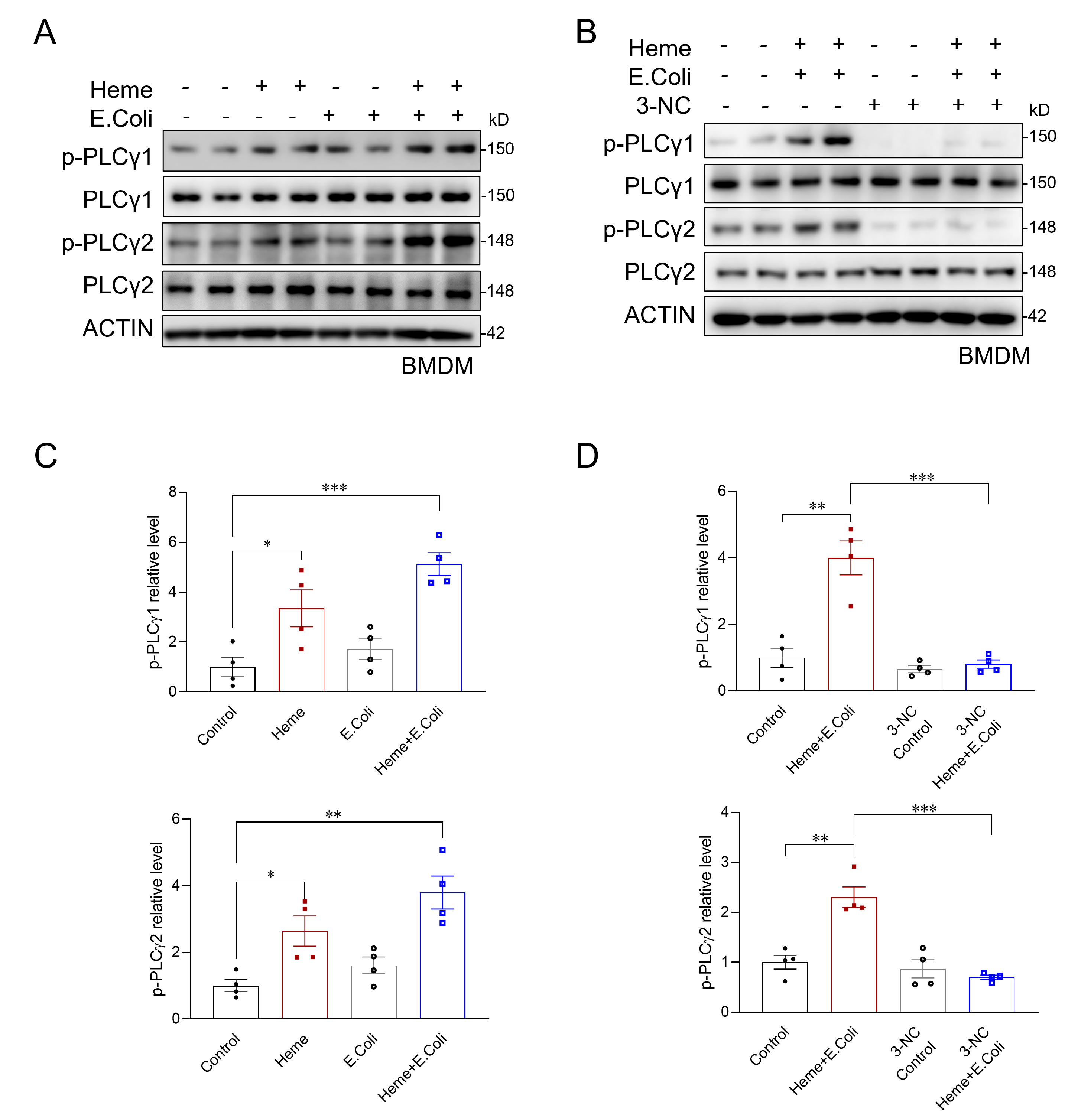


**Figure S1.** **3-NC inhibits Heme-induced PLC-γ activation in macrophages.**
**(A)** Western blot analysis of p-PLCγ1, PLCγ1, p-PLCγ2, and PLCγ2 in BMDMs treated with control, Heme, *E. coli*, or Heme + *E. coli* for 6 hours. **(B)** Western blot analysis of p-PLCγ1, PLCγ1, p-PLCγ2, and PLCγ2 in BMDMs treated with control, Heme + *E. coli*, 3-Nitrocoumarin (3-NC) + control, or 3-NC + Heme + *E. coli* for 3 hours. β-actin was used as a loading control for all western blot analyses. **(C-D)** Quantification of p-PLCγ1 and p-PLCγ2 levels relative to total PLCγ1 and PLCγ2 in BMDMs treated as indicated. (n=4/group). Data are presented as the mean ± SD. *P < 0.05, **P < 0.01, ***P < 0.001. Abbreviations: 3-NC, 3-Nitrocoumarin.


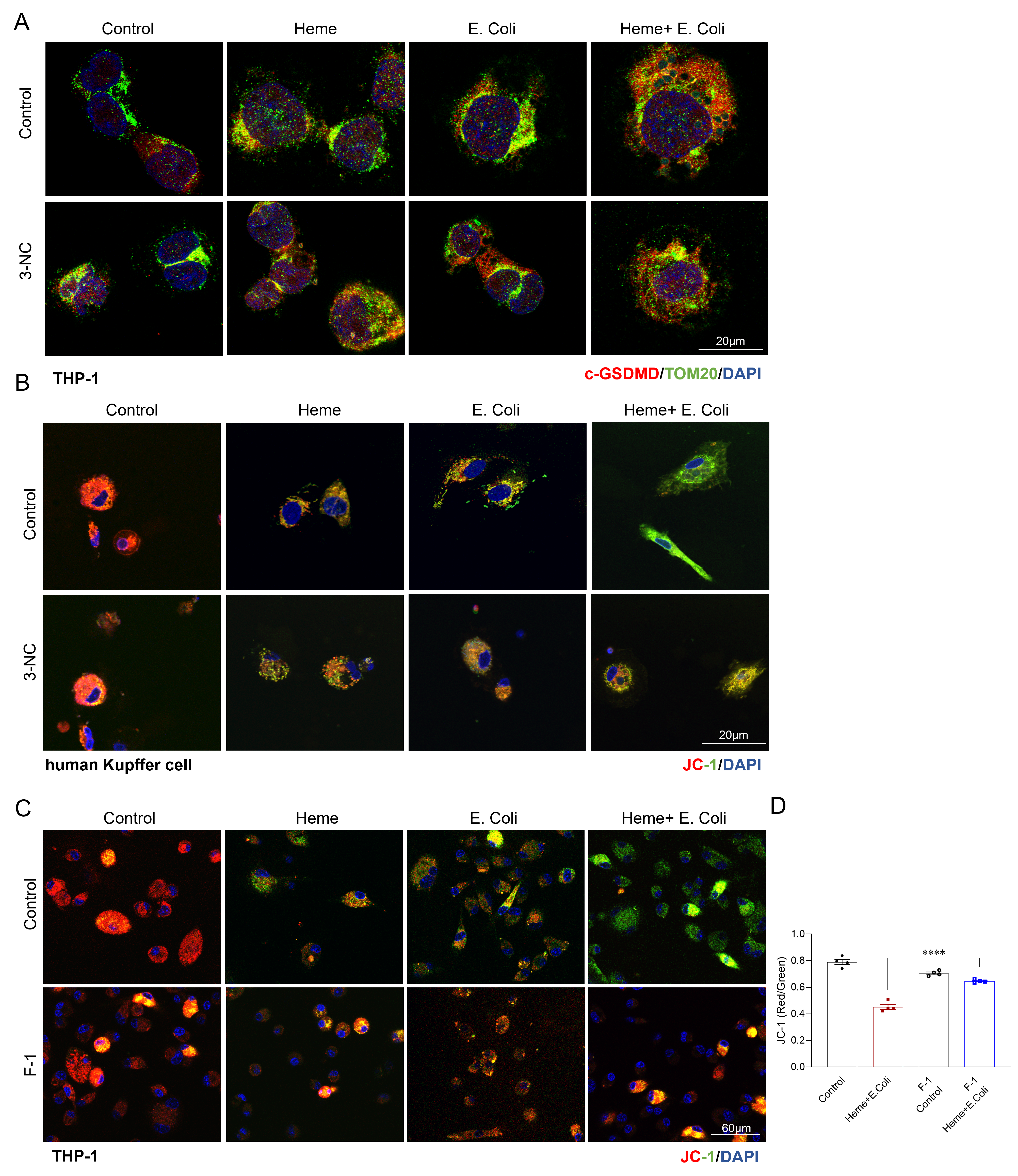


**Figure S2.** **PLC-γ inhibition mitigates mitochondrial damage induced by combined heme and bacterial exposure.**
**(A)** Immunostaining of cleaved GSDMD (c-GSDMD, red) and mitochondrial marker TOM20 (green) in THP-1 cells pre-treated with 3-NC (10 µM) or vehicle control, followed by exposure to Heme, *E. coli*, or Heme + *E. coli* for 3 hours (n=6/group). Scale bars: 20 µm. **(B)** Representative JC-1 staining of human Kupffer cells pre-treated with 3-NC (10 µM) or vehicle control, followed by exposure to Heme, *E. coli*, or Heme + *E. coli* for 3 hours. Scale bars: 20 µm. **(C)** Representative JC-1 staining of live THP-1 cells pre-treated with F-1 (10 µM) or vehicle control, followed by exposure to Heme, *E. coli*, or Heme + *E. coli* for 3 hours (n=4/group). Scale bars: 60 µm. **(D)** Quantitative analysis of the red-to-green fluorescence ratio in JC-1-stained THP-1 cells treated as indicated (n=4/group). Data are presented as mean ± SD. ****P < 0.0001. Abbreviations: 3-NC, 3-Nitrocoumarin; F-1, Ferrostatin-1.


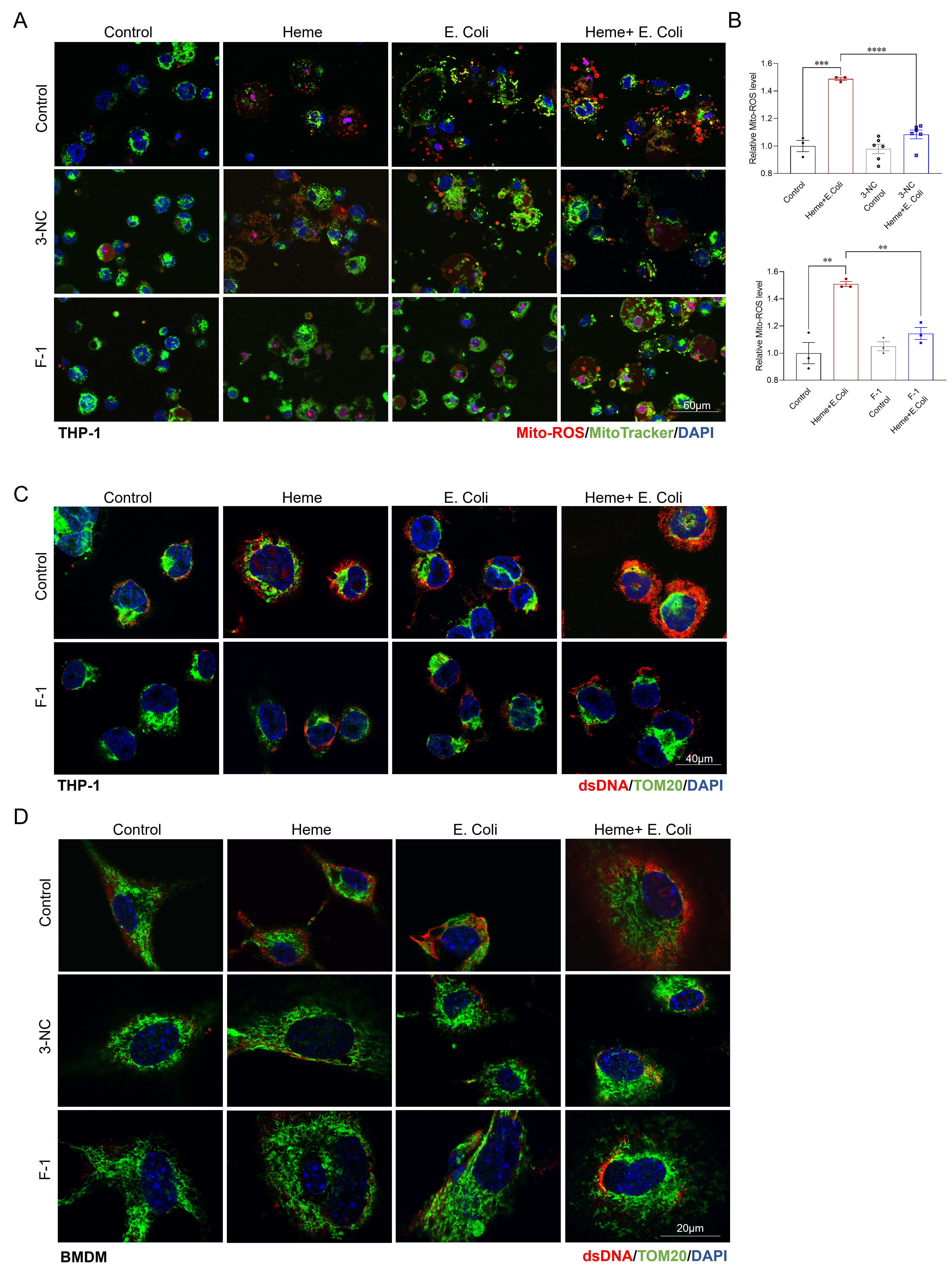


**Figure S3. PLC-γ inhibition reduces mitochondrial ROS and mitigates mitochondrial damage induced by combined heme and bacterial exposure.**
**(A)** Representative images of liver THP-1 cells co-stained for mitochondrial ROS (Mito-SOX, red) and MitoTracker (green) after pre-treated with 3-NC (10 µM), F-1 (10 µM) or vehicle control, followed by exposure to Heme, *E. coli*, or Heme + *E. coli* for 3 hours. (n=6/group). Scale bars: 40 µm. **(B)** Flow cytometry quantification of Mito-ROS levels in THP-1 cells treated as indicated (n=4-6/group). **(C)** Confocal images of THP-1 cells co-stained for double-stranded DNA (dsDNA, red) and mitochondrial marker TOM20 (green) under the indicated conditions (n=6 per group). Scale bars: 20 µm. **(D)** Confocal images of BMDMs co-stained for dsDNA (red) and TOM20 (green) after pre-treatment with 3-NC and F-1 or vehicle control and subsequent exposure to Heme, *E. coli*, or Heme + *E. coli* for 3 hours (n=6/group). Scale bars: 20 µm. Data are presented as mean ± SD. **P < 0.01, ***P < 0.001, ****P < 0.0001.


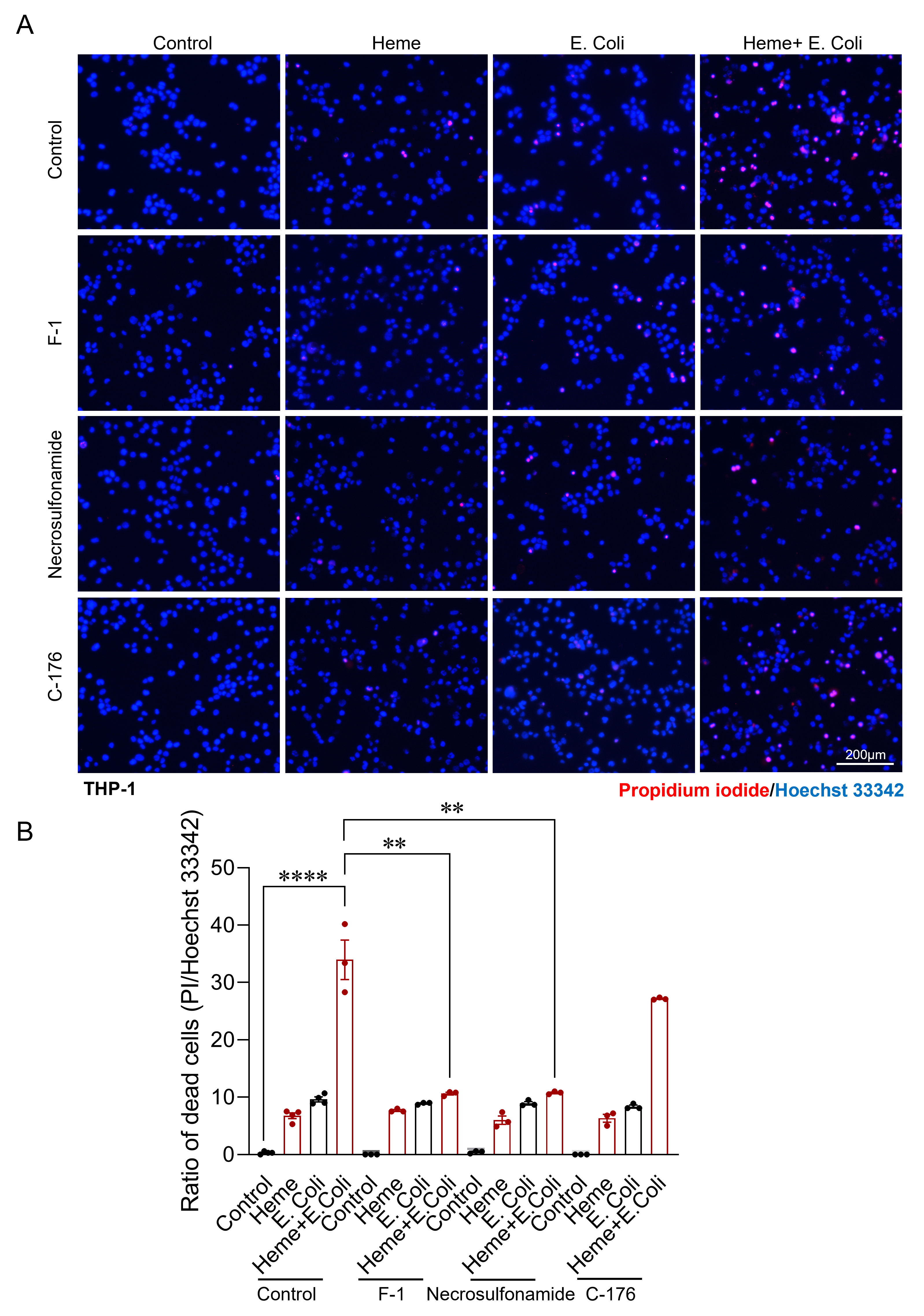


**Figure S4. Ferrostatin-1 (ferroptosis inhibitor) and Necrosulfonamide (Necrosis inhibitor) attenuate cell death induced by combined heme and bacterial exposure.**
**(A)** Representative images of PI staining in THP-1 cells pre-treated with ferroptosis inhibitor (Ferrostatin-1**,**10 µM), necrosis inhibitor (Necrosulfonamide, 10 µM), STING inhibitor (C-176, 2.5 µM), or vehicle control, followed by exposure to Heme, *E. coli*, or Heme + *E. coli* for 24 hours (n=4/group). Scale bars: 200 µm. **(B)** Quantitative analysis of cell death (PI/Hoechst 33342) in THP-1 cells across the indicated groups (n=4/group). Data are presented as mean ± SD. **P < 0.01, ****P < 0.0001. Abbreviations: PI, propidium iodide; F-1, Ferrostatin-1; MLKL, mixed lineage kinase domain-like protein; C-176, STING inhibitor.


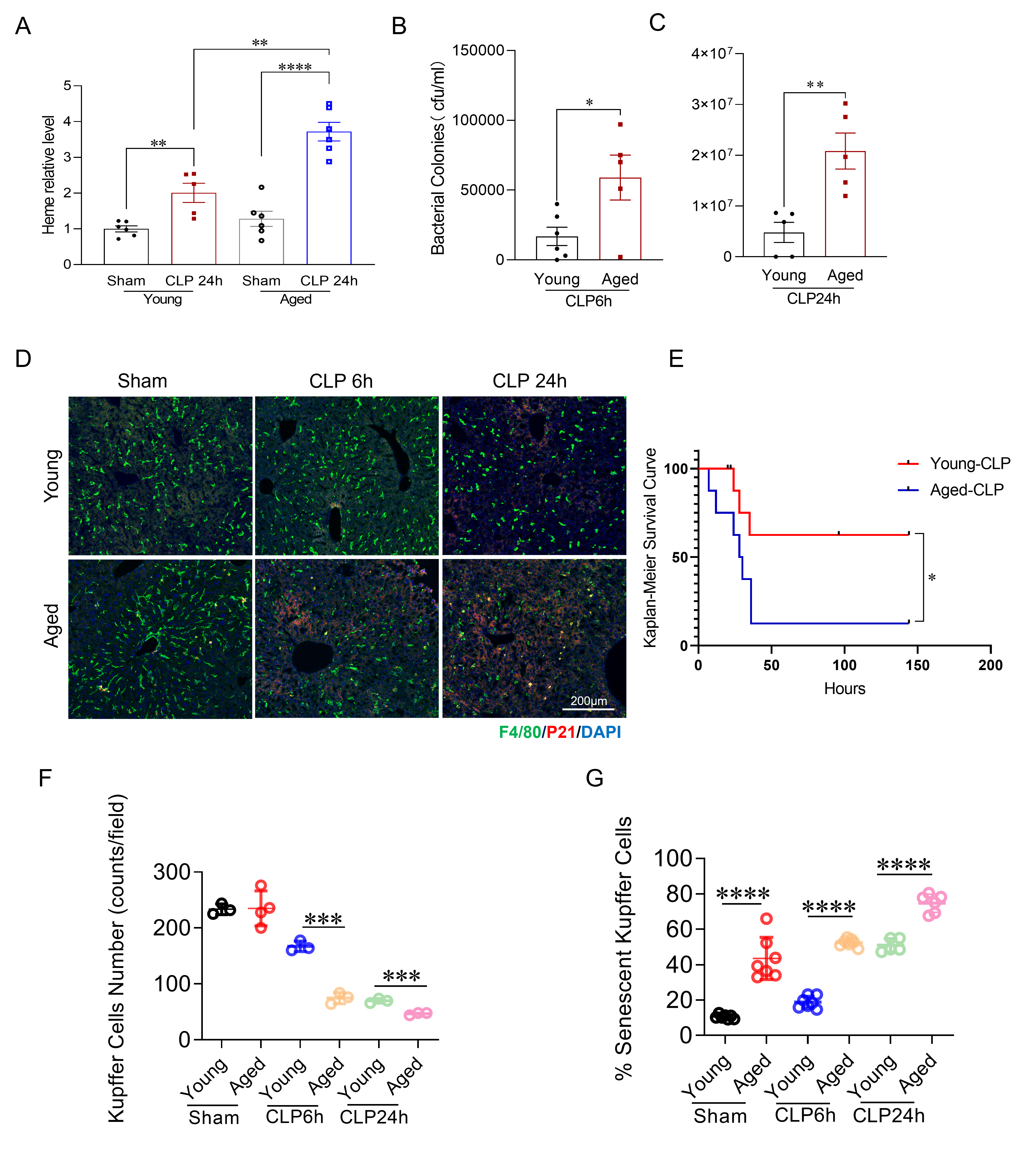


**Figure S5. Aging exacerbates sepsis associated increases in free heme levels, bacterial load, Kupffer cell loss, and mortality. (A)** Quantification of plasma free heme levels in young and aged mice subjected to sham or CLP-induced sepsis (n=6 mice/group). **(B-C)** Quantification of bacterial load in blood from young and aged mice during sepsis (n=5-6 mice/group). **(D)** Representative immunofluorescence staining of F4/80 (green) and p21 (red) in liver sections from young and aged mice subjected to sham or CLP-induced sepsis (n=6 per group). Scale bars: 200 µm. **(E)** Kaplan-Meier survival analysis of young and aged mice subjected to sepsis (n=10/group). **(F-G)** Quantification of Kupffer cell counts and senescent macrophages (F4/80^+^p21^+^) in young and aged mice during sepsis (n=4-8/group). Data are presented as mean ± SD. *P < 0.05, **P < 0.01, ***P < 0.001, ****P < 0.0001. Abbreviations: CLP: Cecal ligation and puncture; F4/80: Macrophage marker; p21: p21^Cip1/Waf1, a senescence marker.


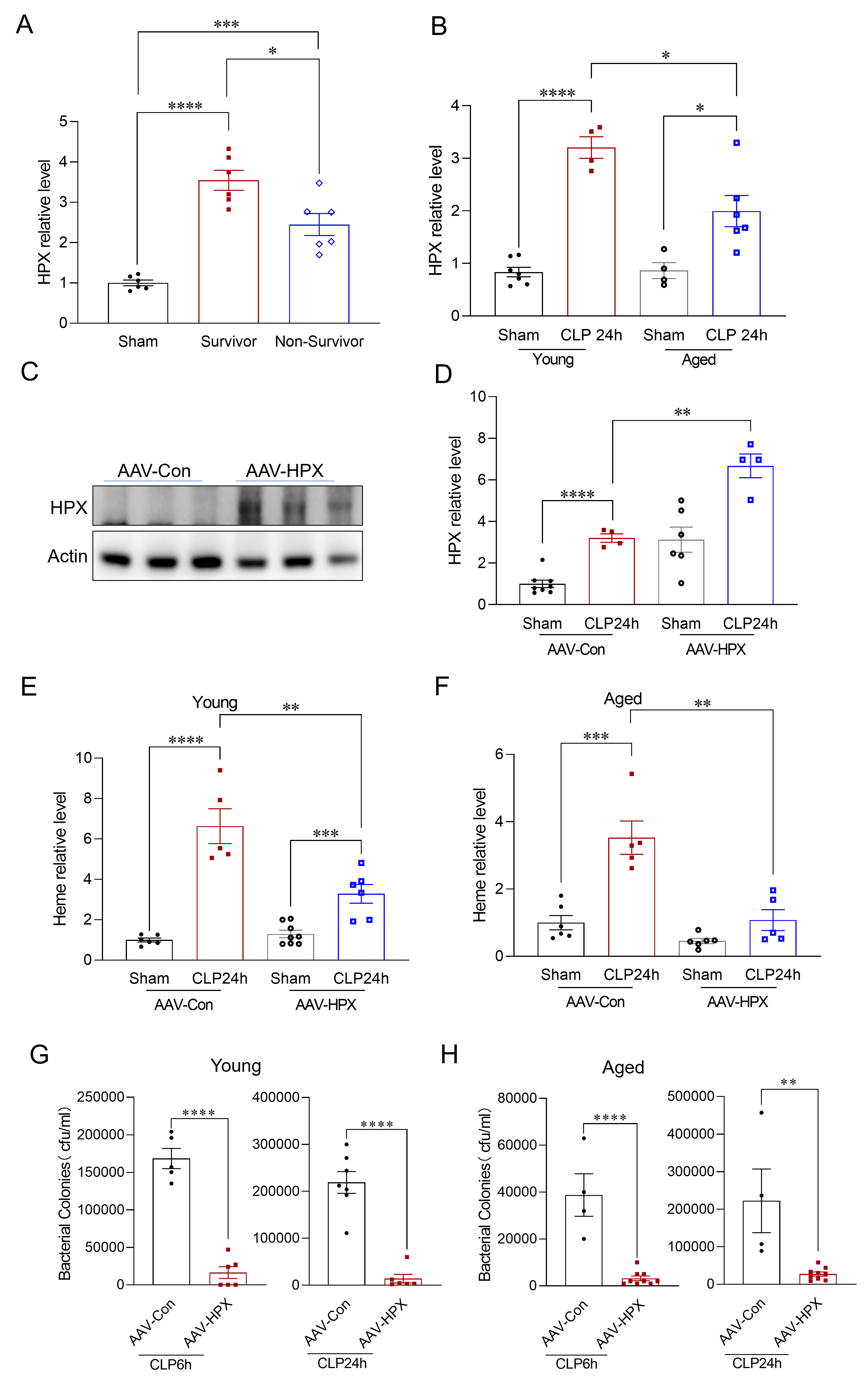


**Figure S6. Increased HPX expression reduces circulating free heme levels and bacterial load in both young and aged mice during sepsis**
**(A)** ELISA quantification of HPX expression in plasma from sham, septic survivors and non-survivors (n=6/group). **(B)** ELISA quantification of HPX expression in plasma from young and aged mice subjected to sham or CLP-induced sepsis (n=4-6/group). **(C)** Western blot analysis of HPX expression in liver tissues from mice administered with AAV-HPX or AAV-Con (n=6/group). β-actin was used as a loading control. **(D)** ELISA quantification of circulating free heme levels in plasma from young and aged mice administered with AAV-HPX or AAV-Con and subjected to sham or CLP-induced sepsis for 24 hours (n=4-6/group). **(E-F)** Quantification of circulating free heme levels relative to sham in young and aged mice administered with AAV-HPX or AAV-Con and subjected to CLP-induced sepsis for 24 hours (n=6-8/group). **(G-H)** Quantification of bacterial load in blood from young and aged mice administered with AAV-HPX or AAV-Con and subjected to sham or CLP-induced sepsis for 24 hours (n=4-9/group). Data are presented as mean ± SD. *P < 0.05, **P < 0.01, ***P < 0.001, ****P < 0.0001. Abbreviations: HPX: Hemopexin; AAV-HPX: Adeno-associated virus encoding HPX; AAV-Con: Adeno-associated virus control; CLP: Cecal ligation and puncture


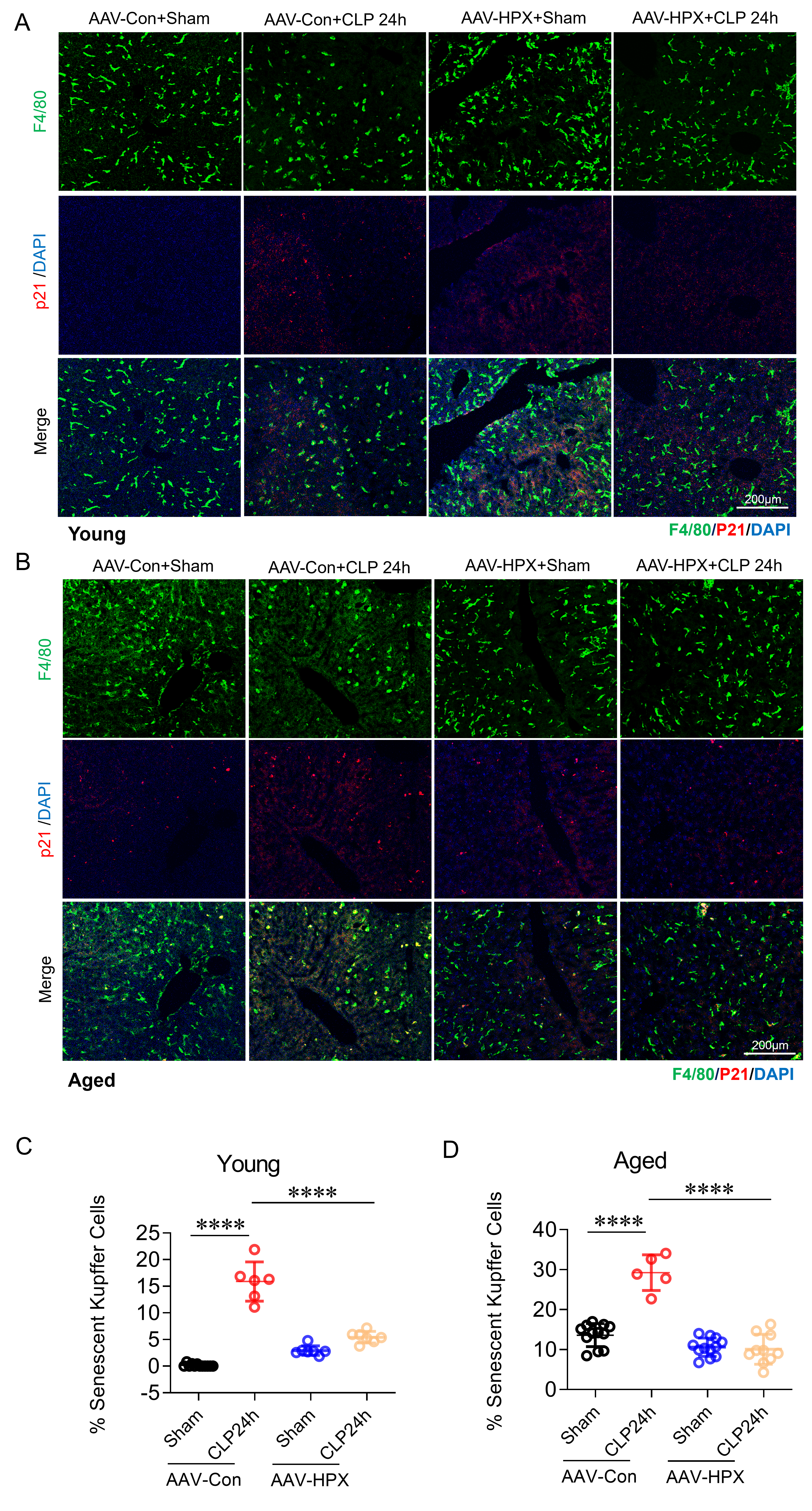


**Figure S7. Increased HPX expression alleviates Kupffer cell senescence during sepsis in young and aged mice**
**(A-B)** Representative immunofluorescence staining of p21 (red) and F4/80 (green) in liver sections from young and aged mice administered with AAV-HPX or AAV-Con and subjected to CLP sepsis (n=6/group). Scale bars: 200 µm. **(C-D)** Quantification of senescent Kupffer cells (F4/80^+^p21^+^) in young and aged mice across the indicated treatment groups (n=6-10/group). Data are presented as mean ± SD. ****P < 0.0001.
