# Supplementary material for: The role of heme in sepsis induced Kupffer cell PANoptosis and senescence
Source: Cell Death Dis. 2025 Apr 13;16(1):284. doi: 10.1038/s41419-025-07637-6 (PMC11993645; doi:10.1038/s41419-025-07637-6)

Figure.2 Heme administration exacerbates sepsis-induced Kupffer cell loss, senescence, bacterial load, and mortality.

E

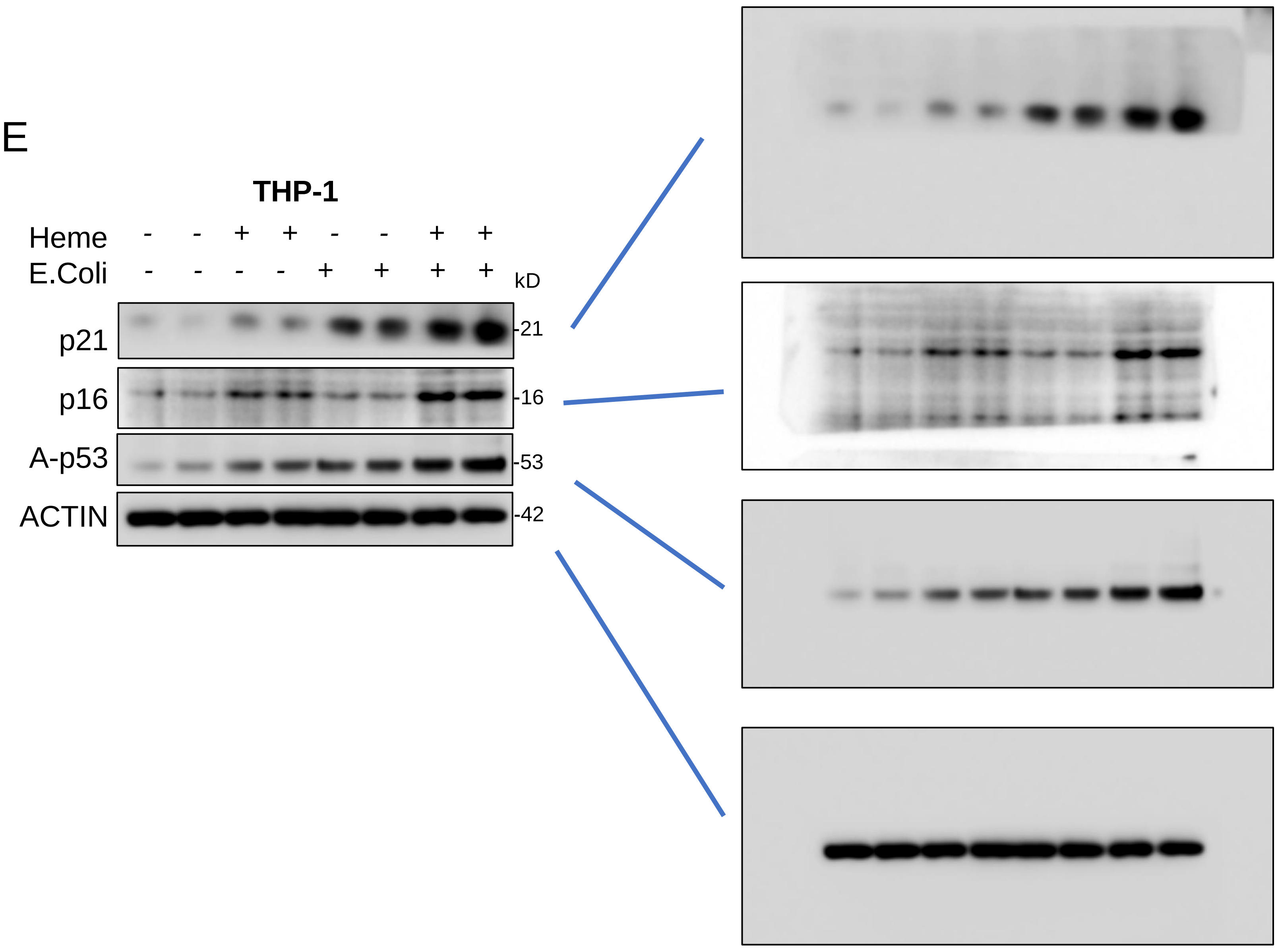

F

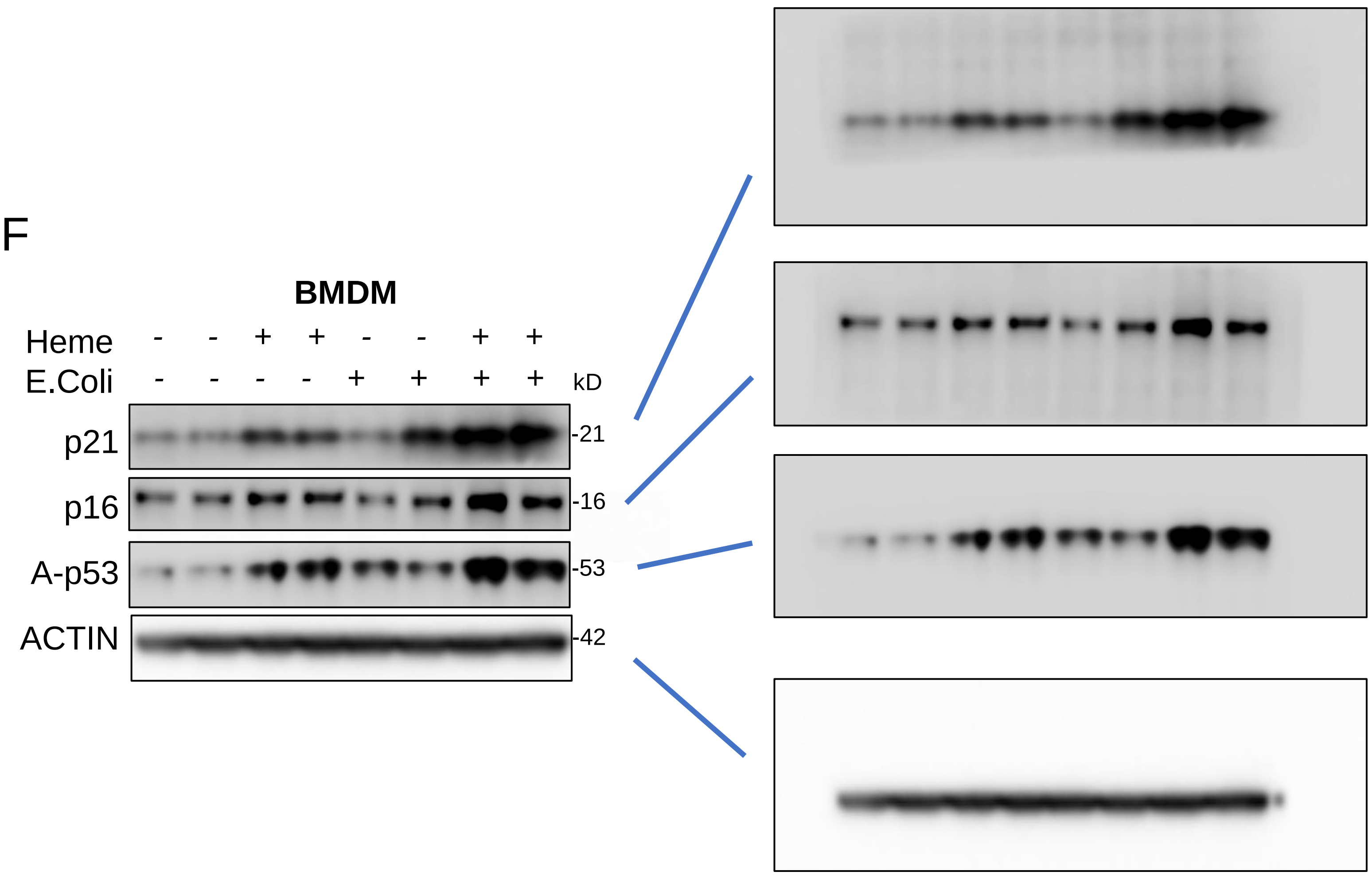

Figure.3 Heme exacerbates bacterial-induced PANoptosis in macrophages.

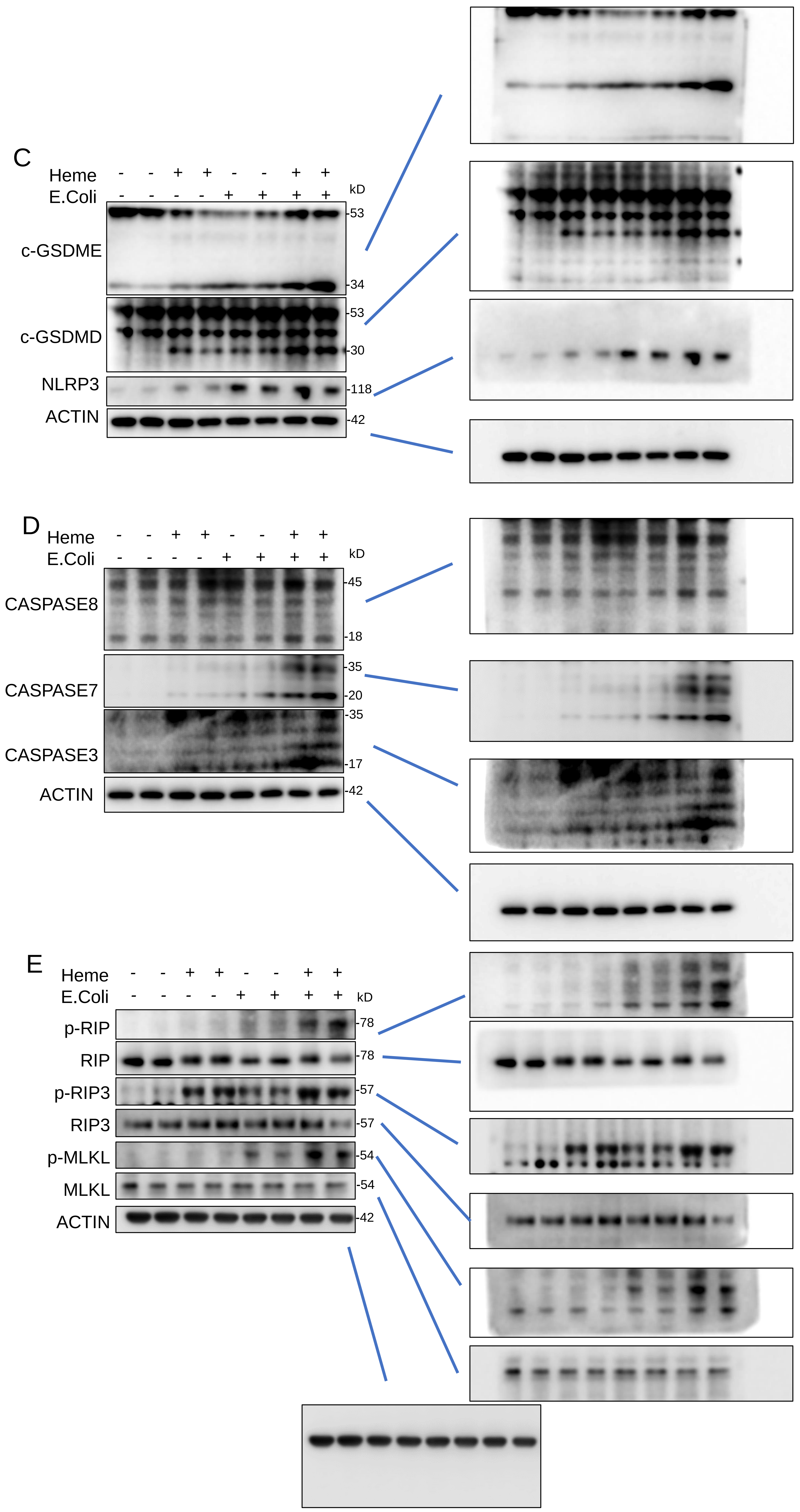

Figure.3 Heme exacerbates bacterial-induced PANoptosis in macrophages.

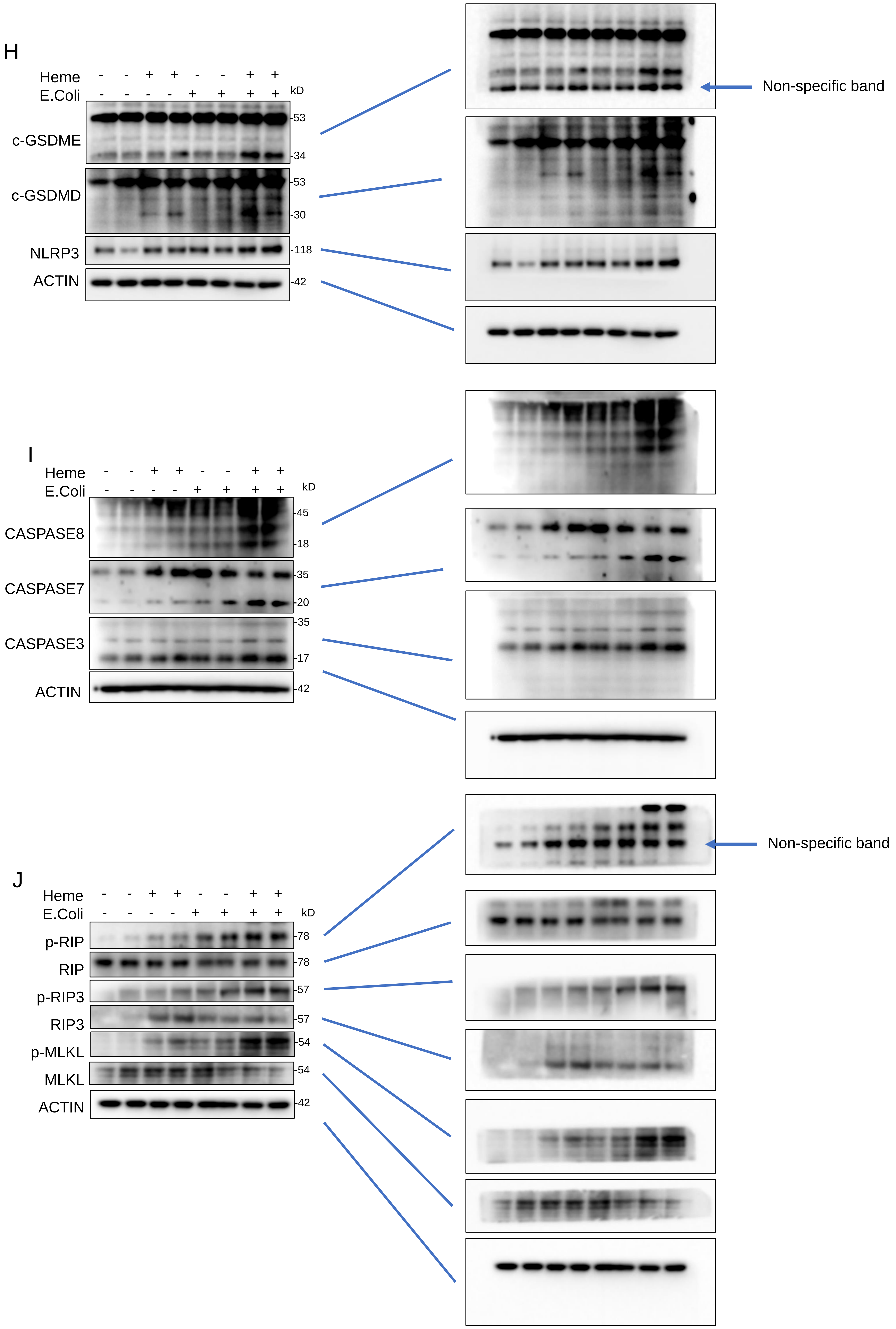

Figure.5 PLC-γ mediated GSDMD mitochondrial translocation contributes to combined heme and bacterial induced mitochondrial damage

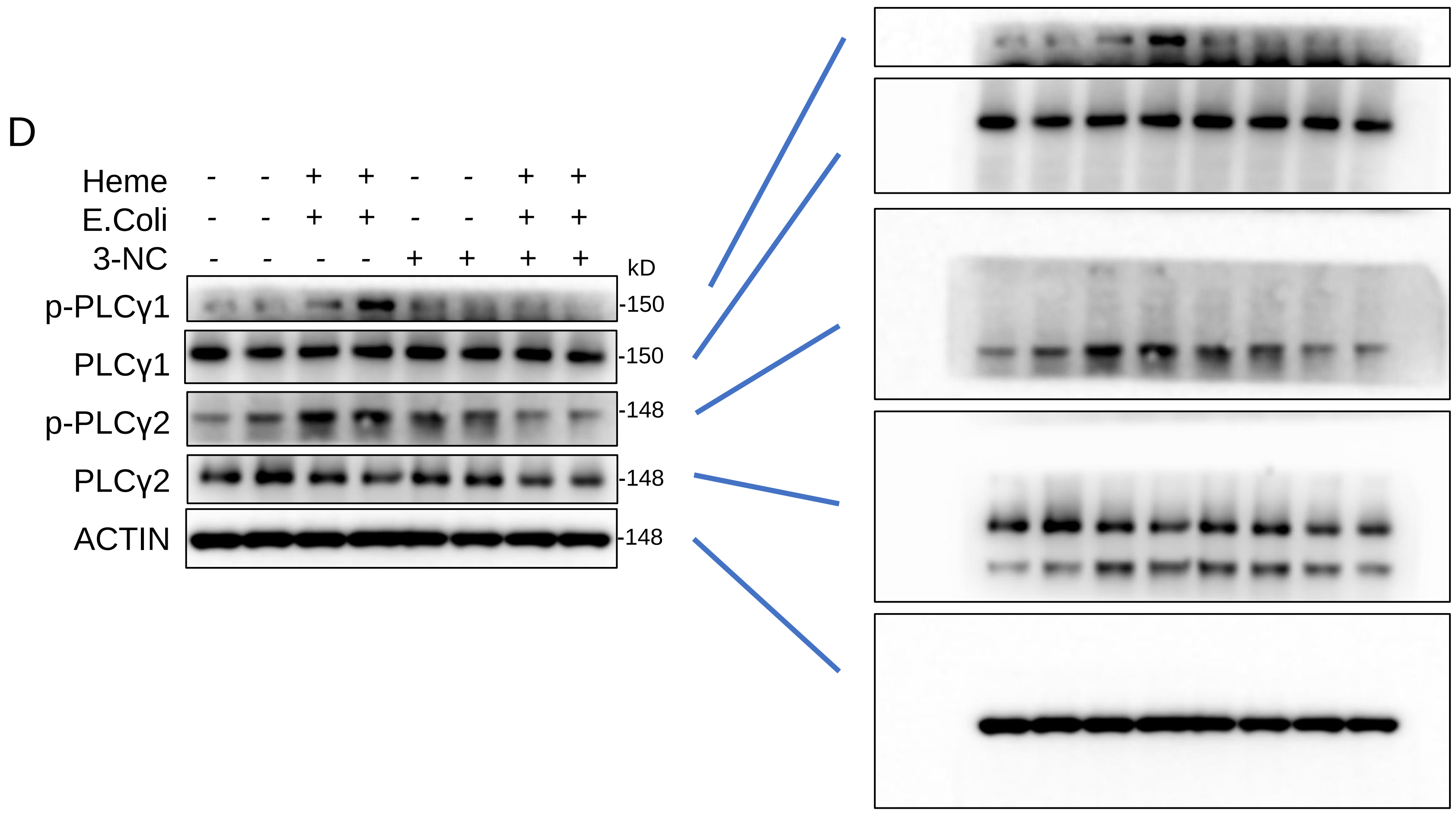

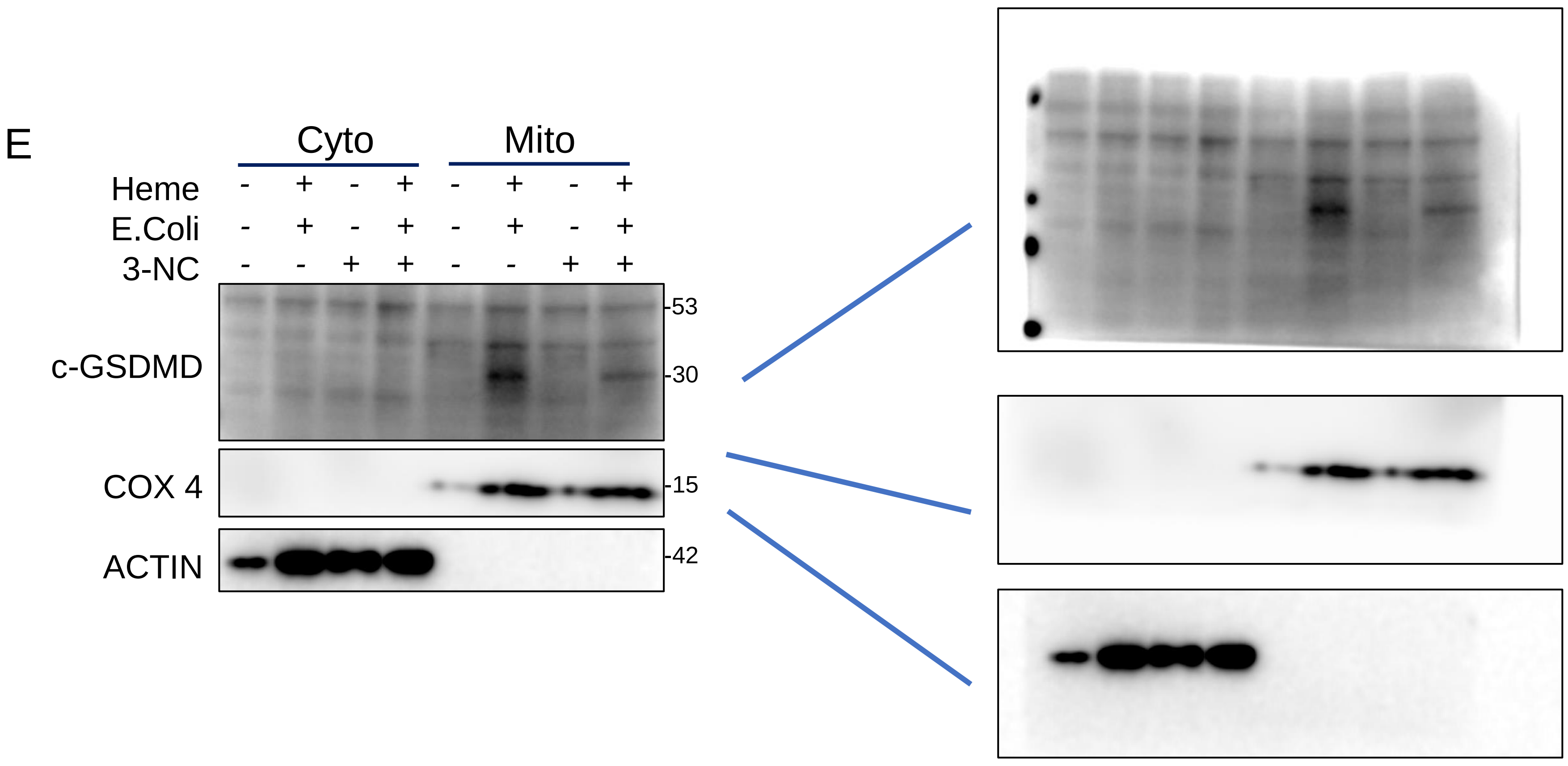

Figure.5 PLC-γ mediated GSDMD mitochondrial translocation contributes to combined heme and bacterial induced mitochondrial damage

A

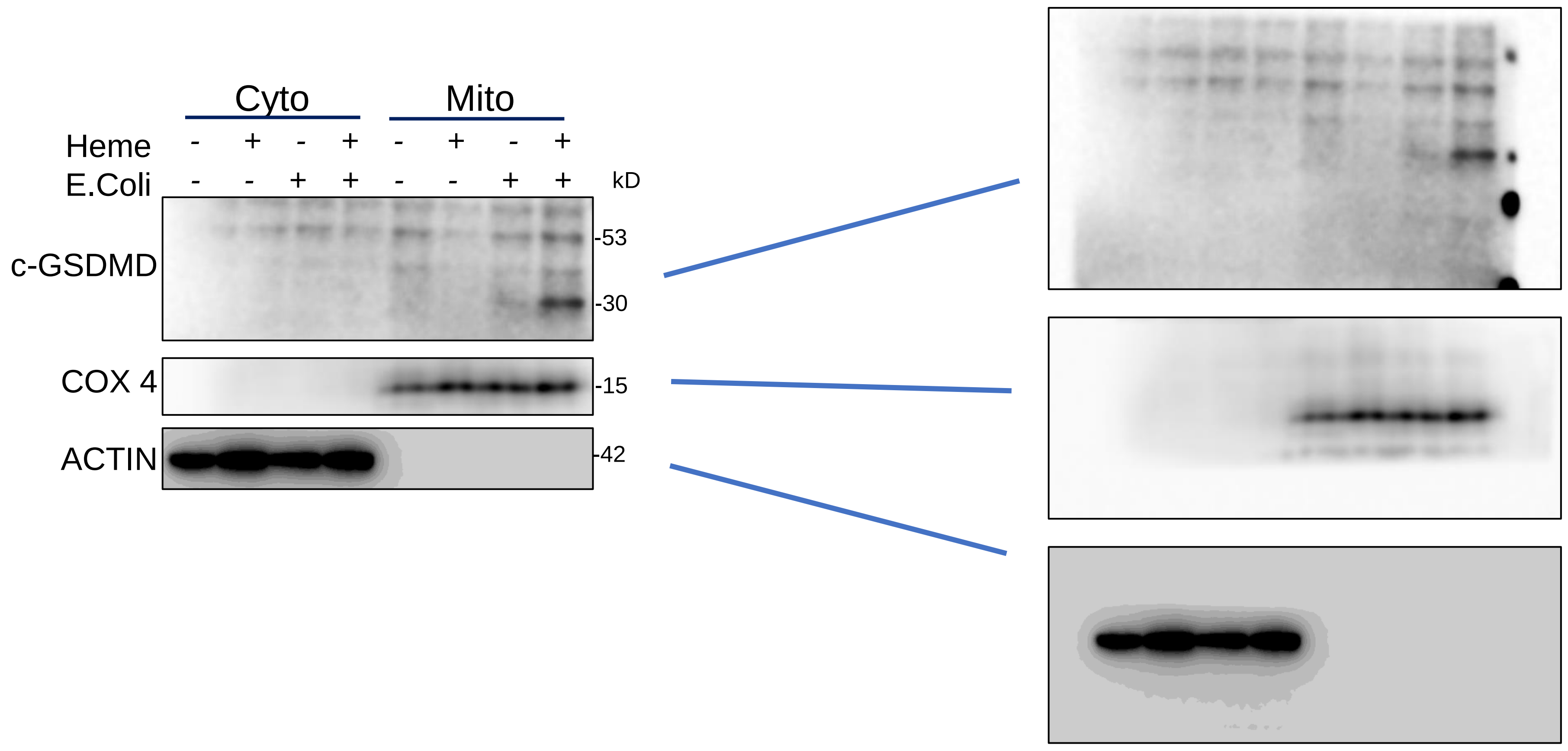

B

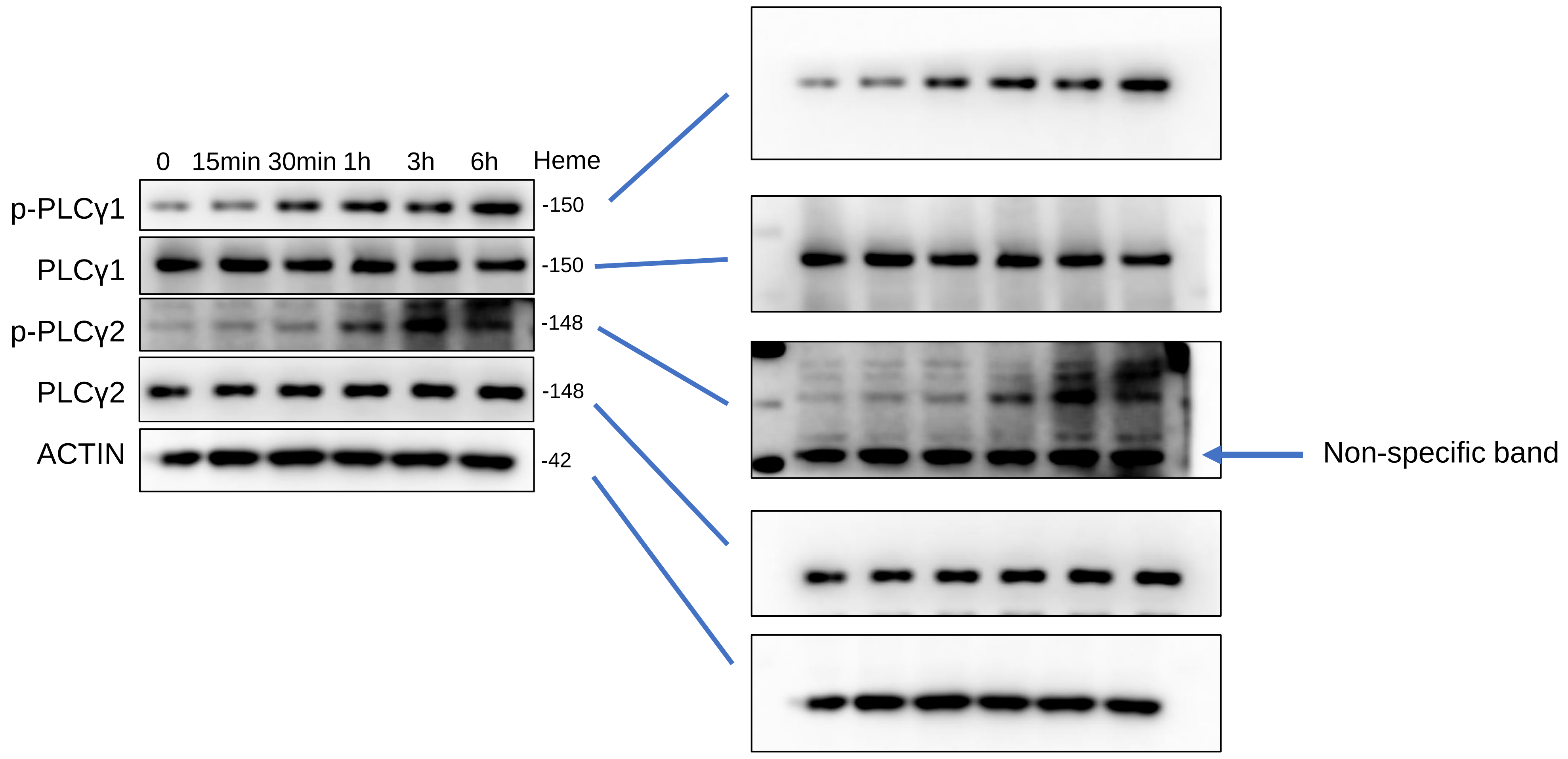

C

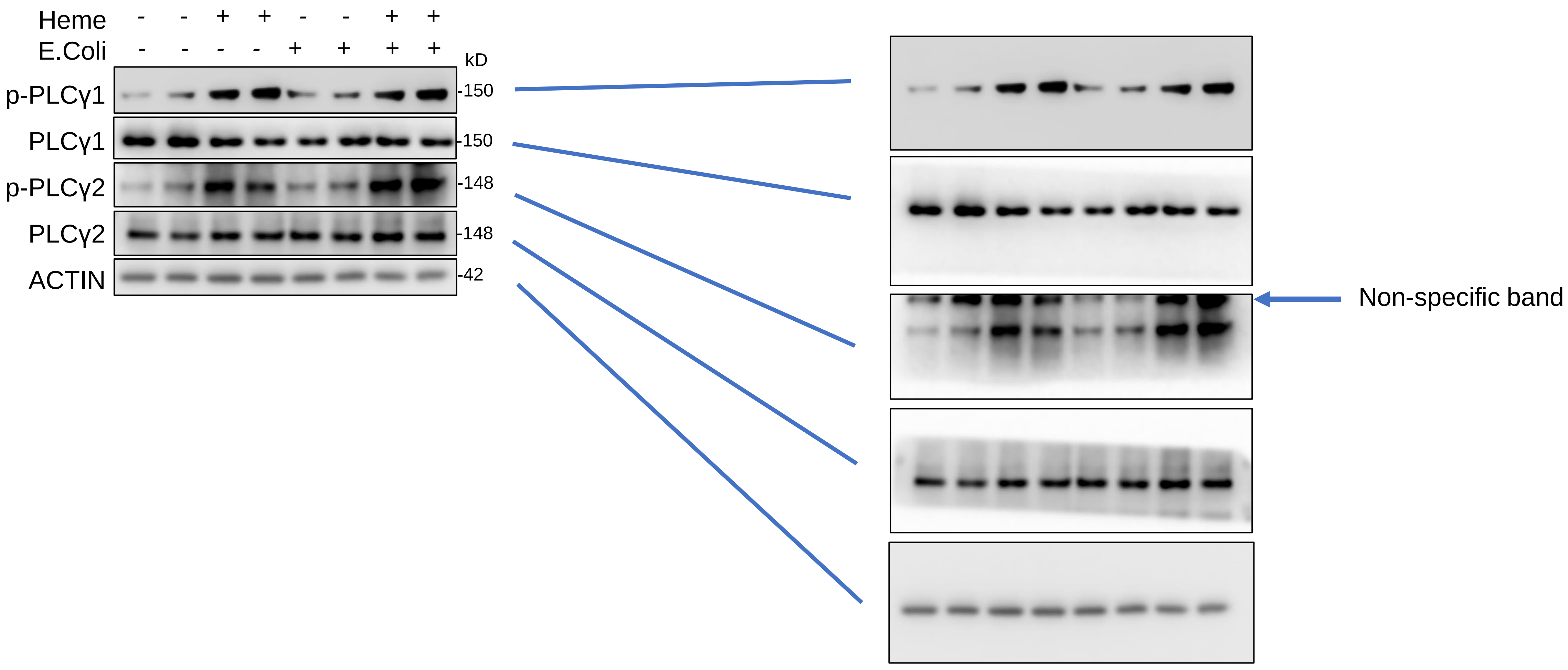

Figure.6 PLC-γ inhibition mitigates combined heme and bacterial induced macrophage PANoptosis

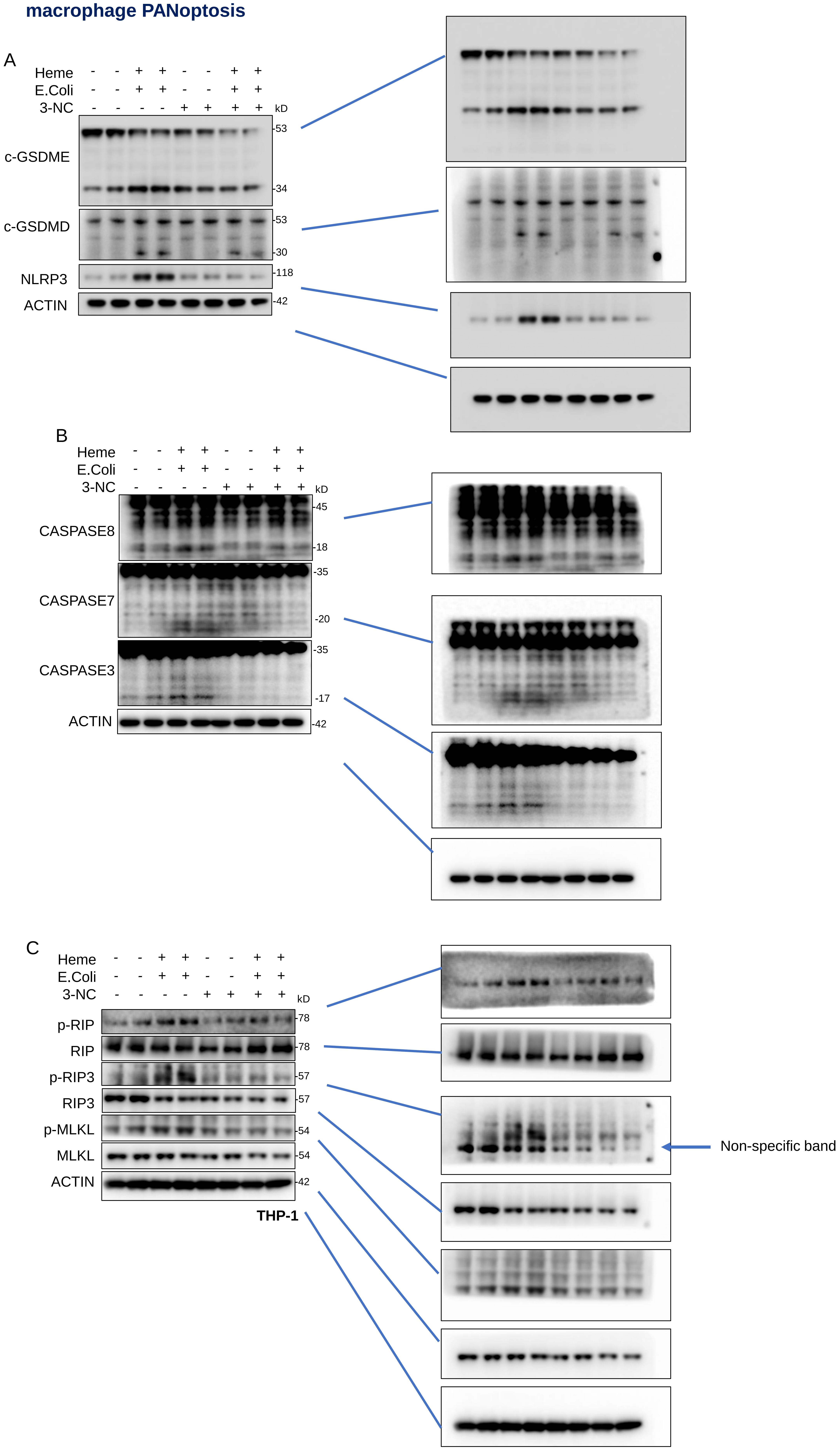

Figure.6 PLC-γ inhibition mitigates combined heme and bacterial induced macrophage PANoptosis

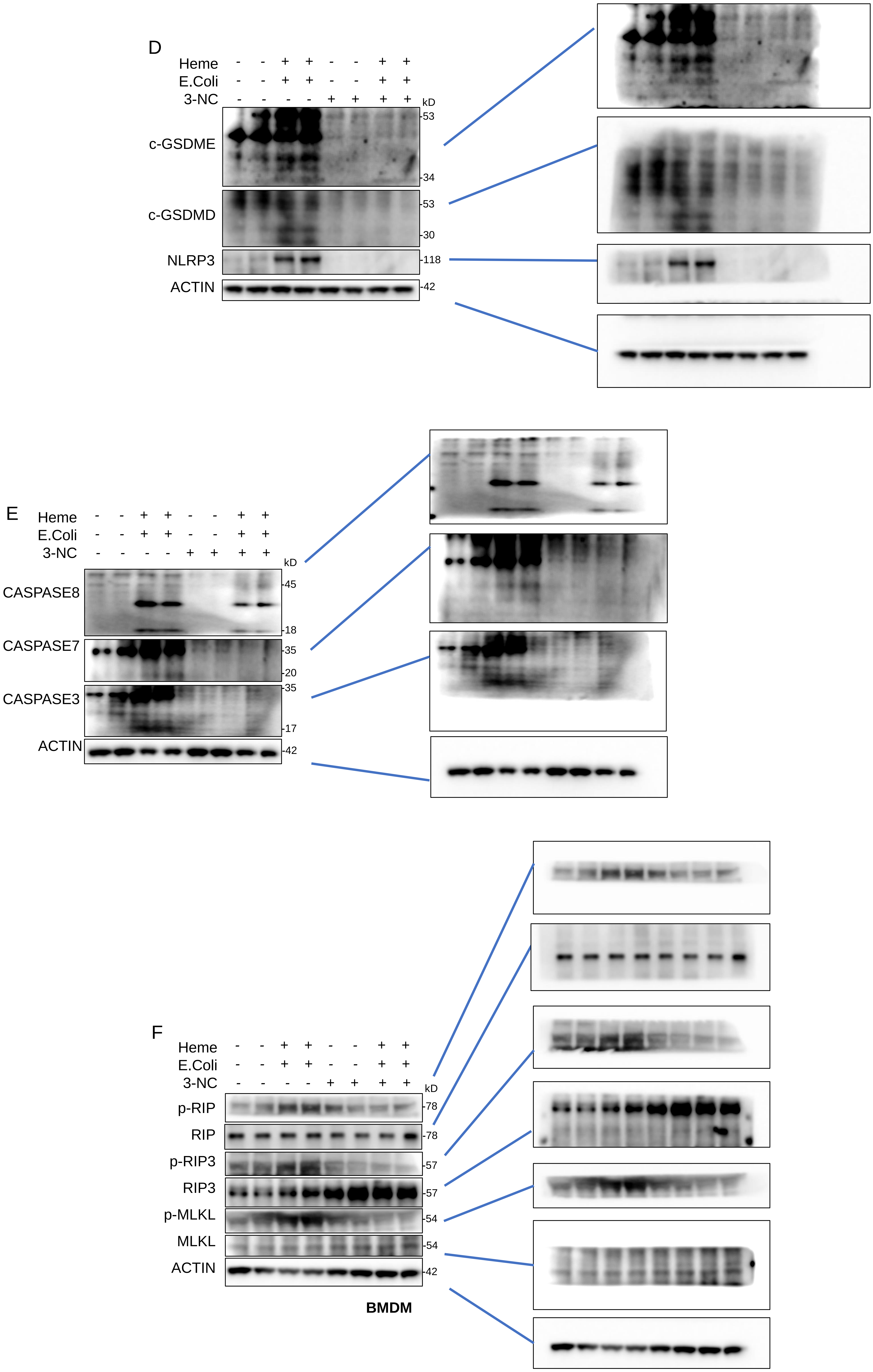

Figure.7 cGAS-STING activation contributes to combined heme and bacterial induced macrophage senescence.

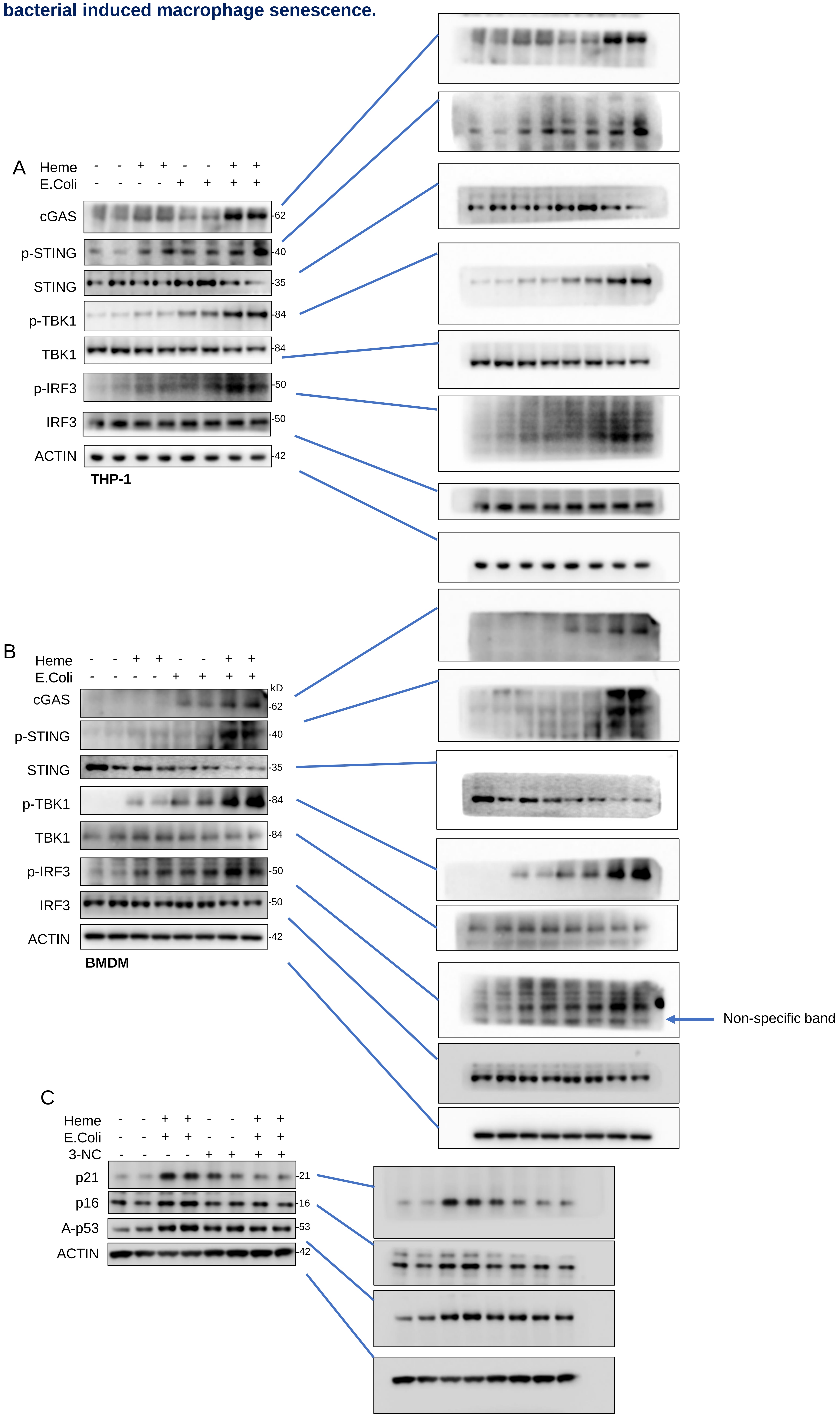

Figure.7 cGAS-STING activation contributes to combined heme and bacterial induced macrophage senescence.

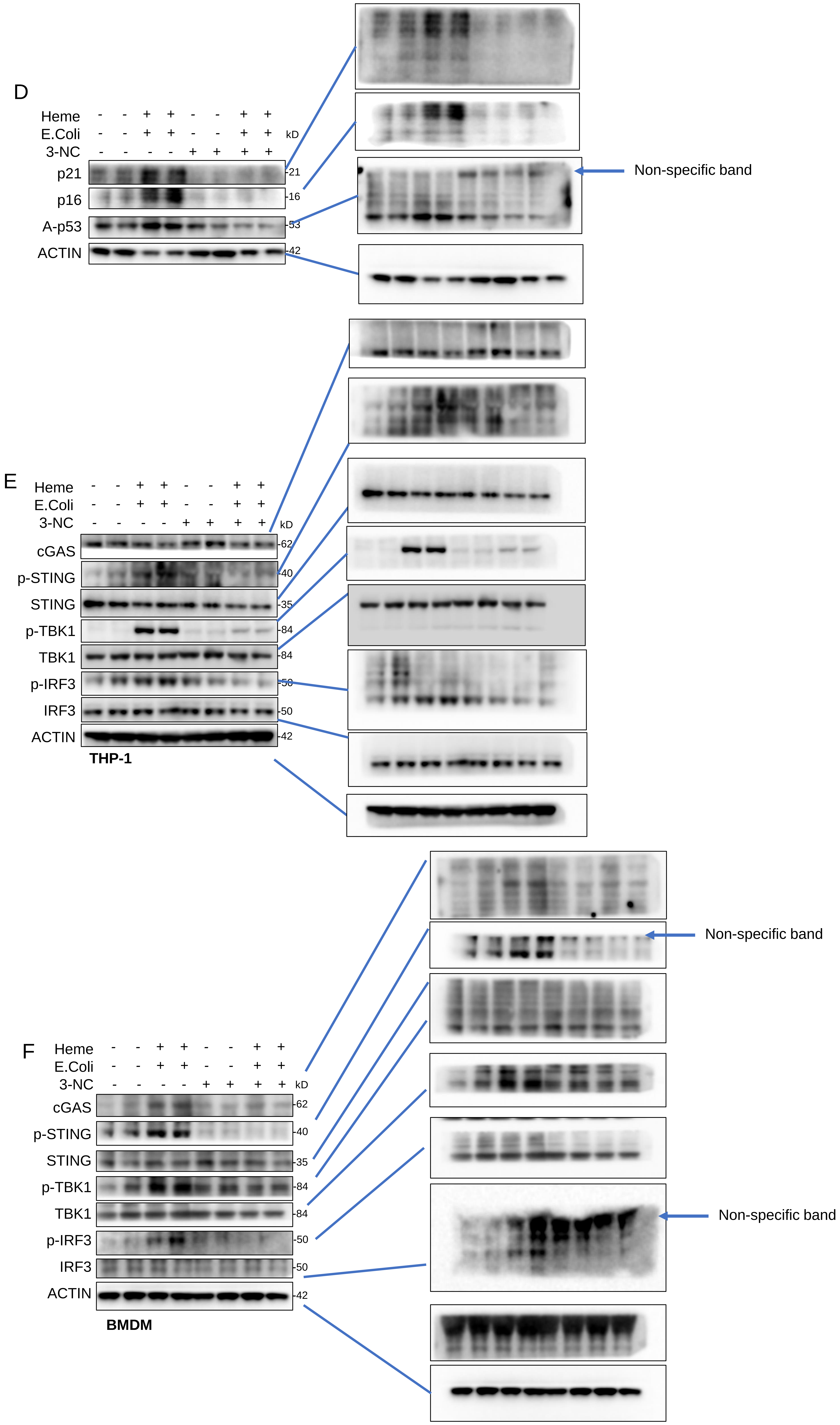

Figure.7 cGAS-STING activation contributes to combined heme and bacterial induced macrophage senescence.

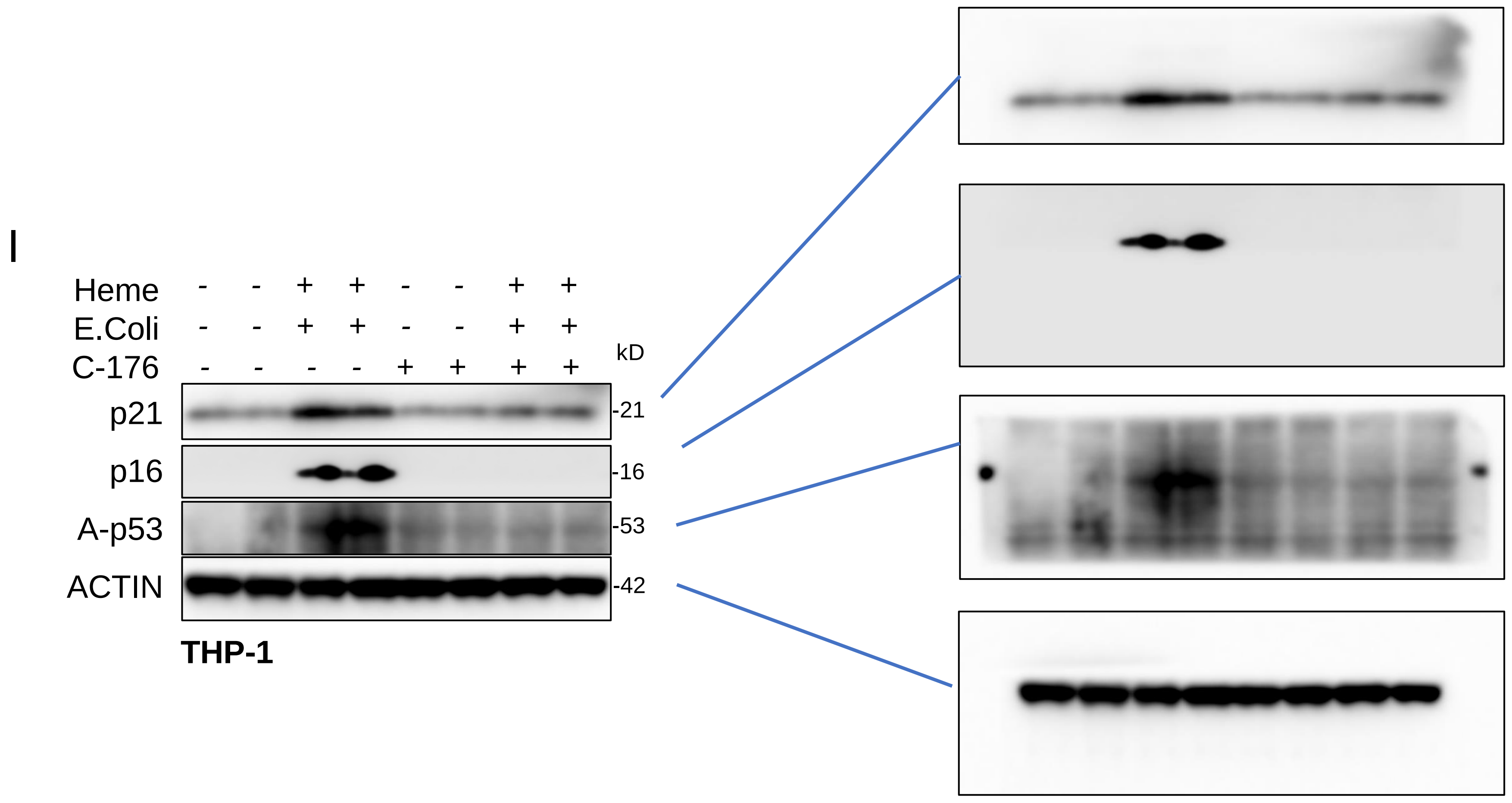

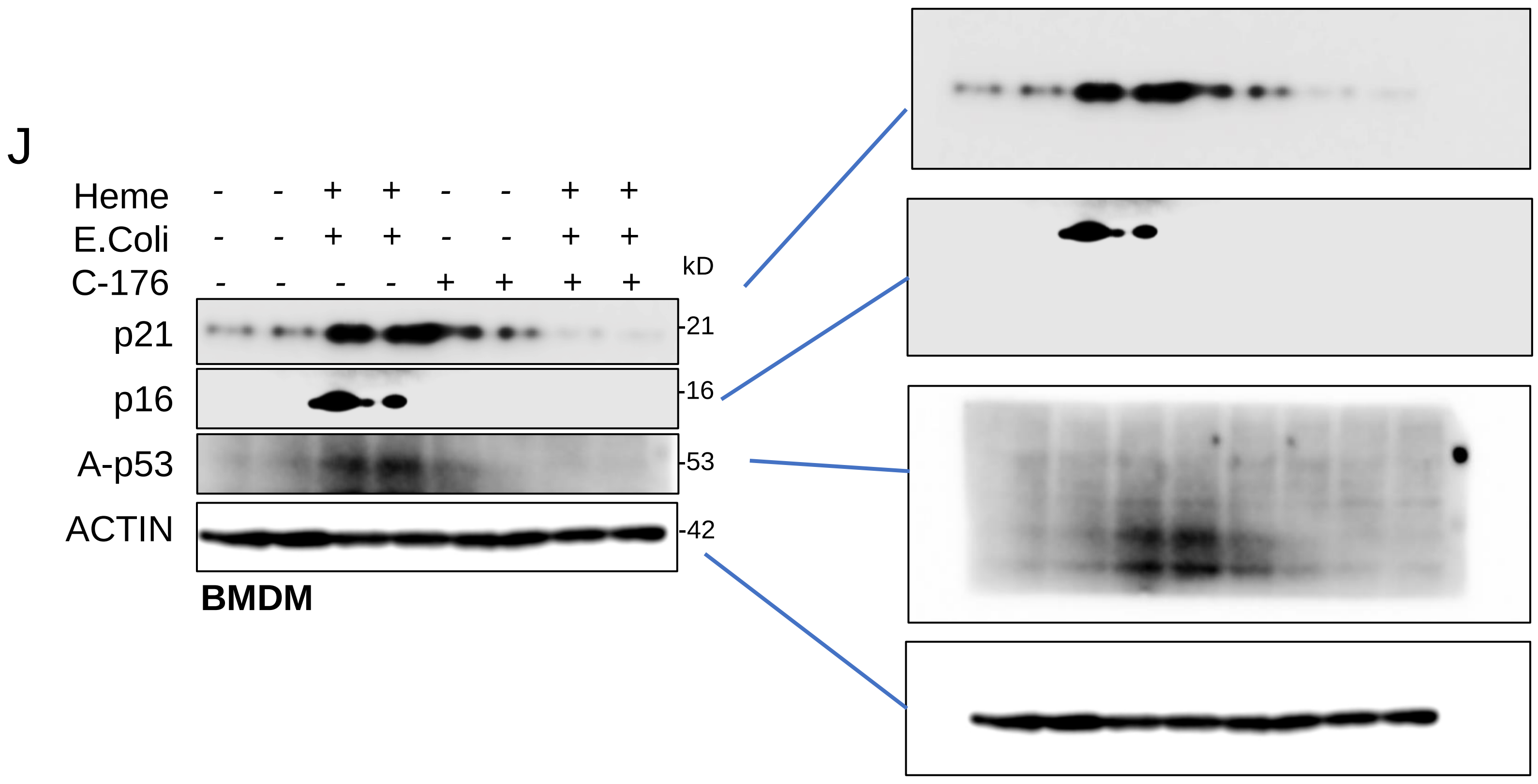

Figure. 8 Increased hemopexin expression alleviates sepsis-induced Kupffer cell loss and improves survival in both young and aged mice.

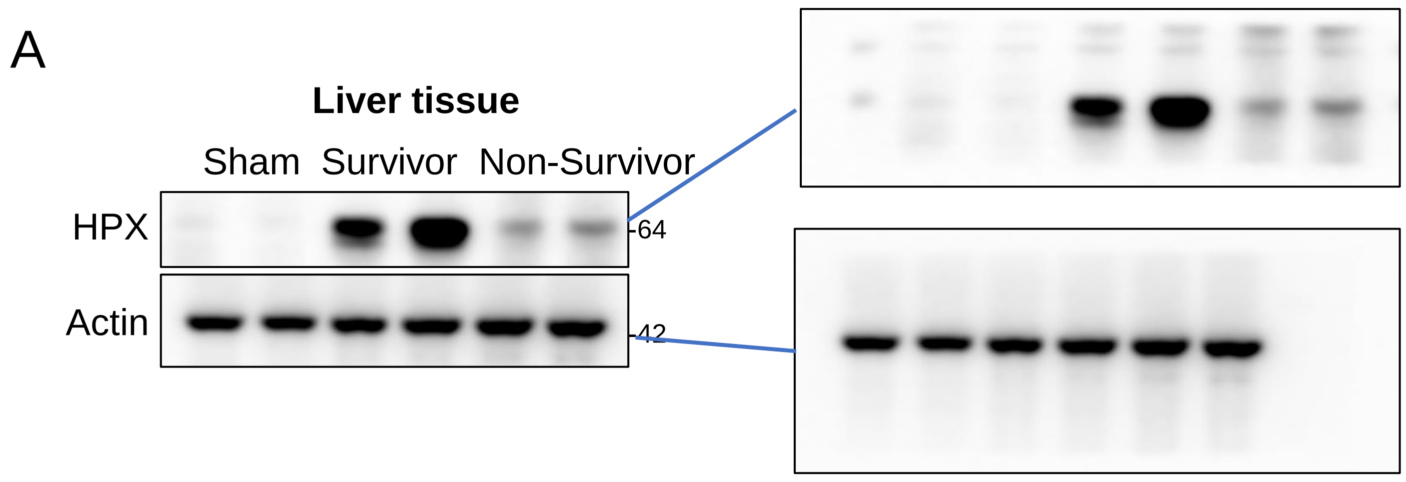

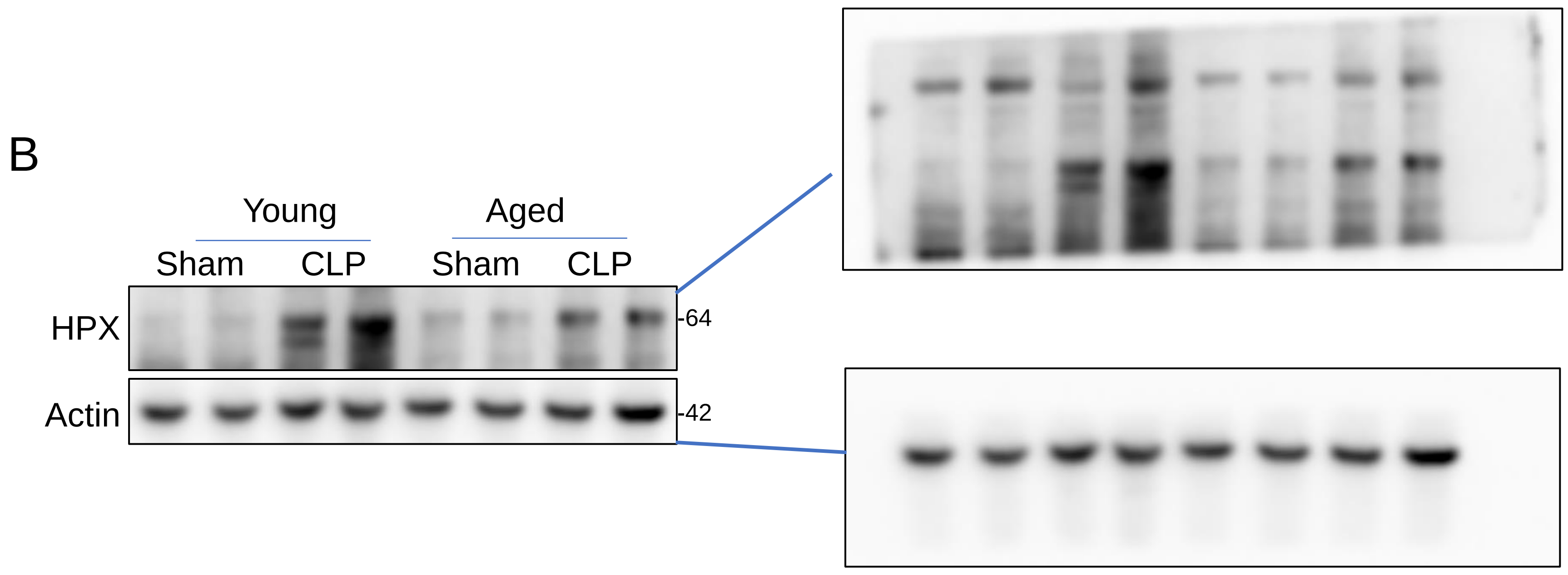

Figure. S1

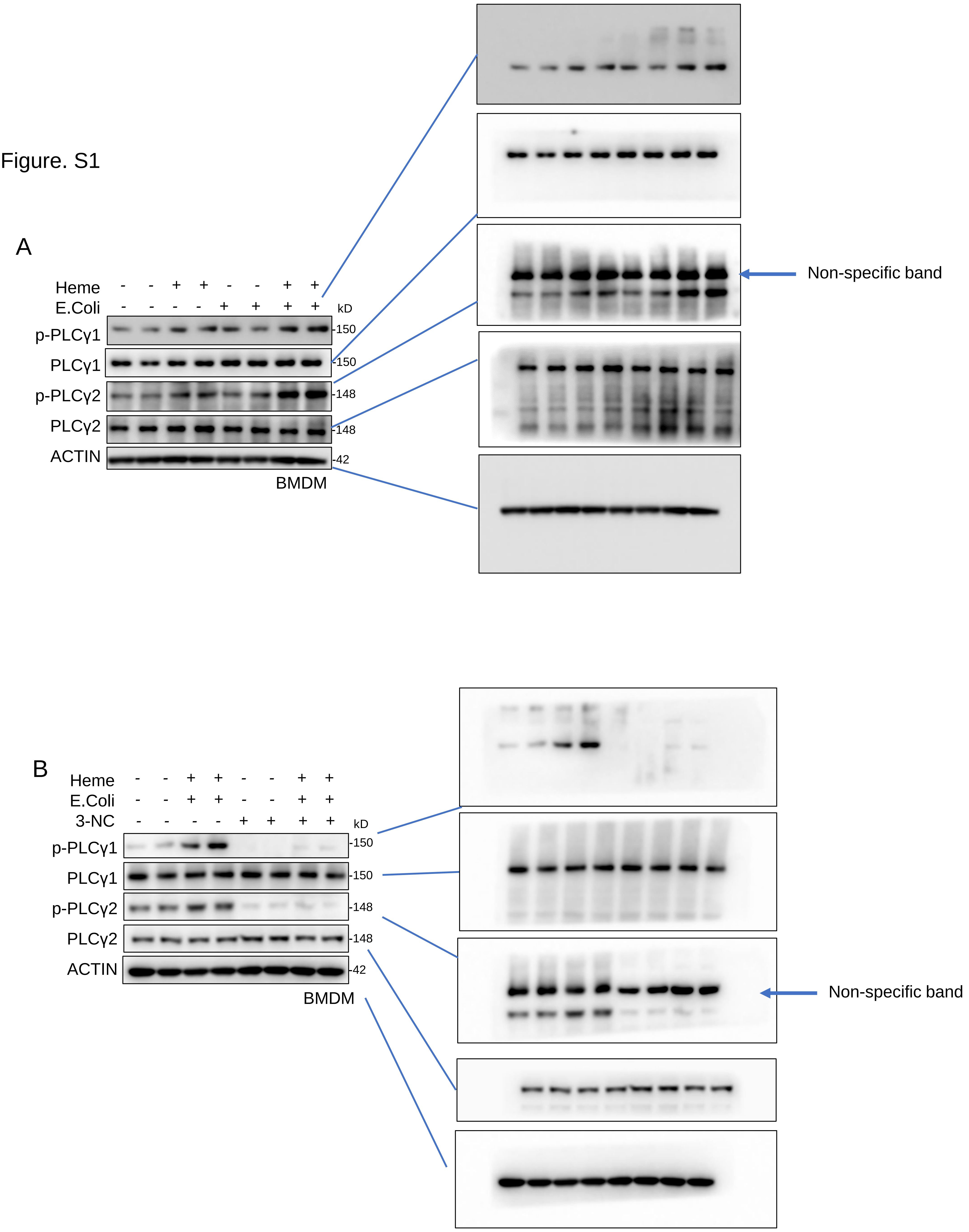

Figure. S6

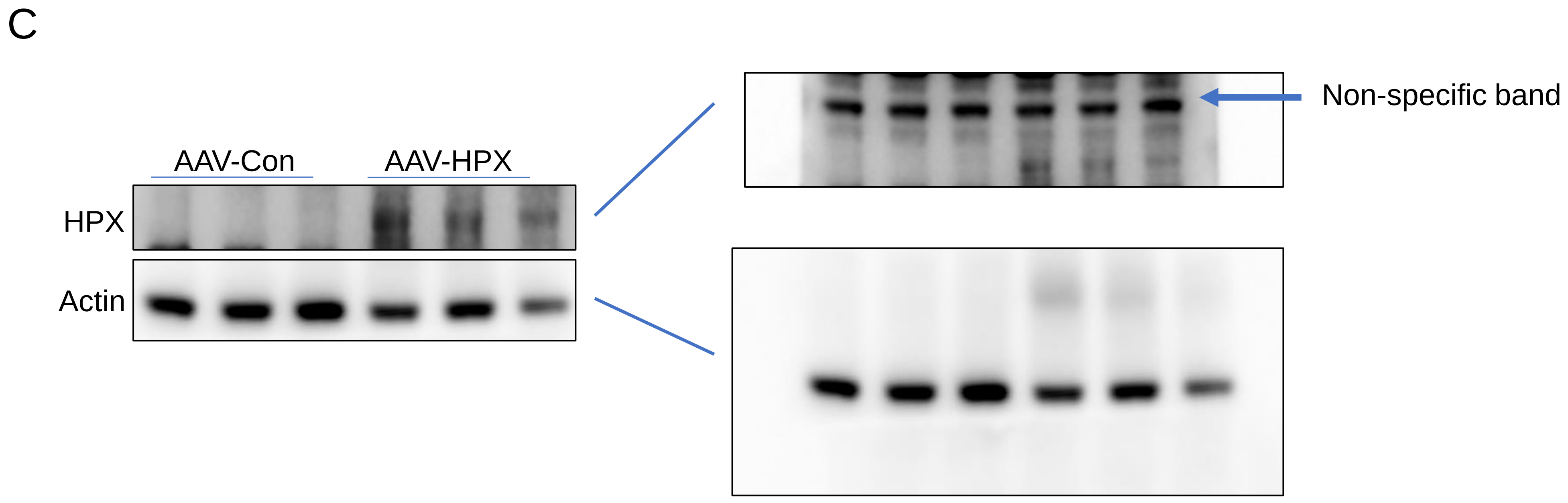

Supplement: Supplementary file 2 — Full and uncropped western blots [file 41419_2025_7637_MOESM2_ESM.pdf]
